# The Nitrite Transporter Facilitates Biofilm Formation via Suppression of Nitrite Reductase and Is a New Antibiofilm Target in Pseudomonas aeruginosa

**DOI:** 10.1128/mBio.00878-20

**Published:** 2020-07-07

**Authors:** Ji-Su Park, Ha-Young Choi, Won-Gon Kim

**Affiliations:** a Infectious Disease Research Center, Korea Research Institute of Bioscience and Biotechnology, Daejeon, Republic of Korea; b Department of Bio‑Molecular Science, KRIBB School of Bioscience, Korea University of Science and Technology (UST), Daejeon, Republic of Korea; Emory University School of Medicine

**Keywords:** biofilms, *Pseudomonas aeruginosa*, nitric oxide, nitrite transporter, drug target

## Abstract

Biofilm-forming bacteria, including the Gram-negative Pseudomonas aeruginosa, cause multiple types of chronic infections and are responsible for serious health burdens in humans, animals, and plants. Nitric oxide (NO) has been shown to induce biofilm dispersal via triggering a reduction in cyclic-di-GMP levels in a variety of bacteria. However, how NO, at homeostatic levels, also facilitates biofilm formation is unknown. Here, we found that complestatin, a structural analog of vancomycin isolated from *Streptomyces*, inhibits P. aeruginosa biofilm formation by upregulating NO production via nitrite reductase (NIR) induction and c-di-GMP degradation via phosphodiesterase (PDE) stimulation. The complestatin protein target was identified as a nitrite transporter from a genome-wide screen using the Keio Escherichia coli knockout library and confirmed using nitrite transporter knockout and overexpression strains. We demonstrated that the nitrite transporter stimulated biofilm formation by controlled NO production via appropriate NIR suppression and subsequent diguanylate cyclase (DGC) activation, not PDE activity, and c-di-GMP production in E. coli and P. aeruginosa. Thus, this study provides a mechanism for NO-mediated biofilm formation, which was previously not understood.

## Abstract (precis)

Bacterial biofilms play roles in infections and avoidance of host defense mechanisms of medically important pathogens and increase the antibiotic resistance of the bacteria. Nitric oxide (NO) is reported to be involved in both biofilm formation and dispersal, which are conflicting processes. The mechanism by which NO regulates biofilm dispersal is relatively understood, but there are no reports about how NO is involved in biofilm formation. Here, by investigating the mechanism by which complestatin inhibits biofilm formation, we describe a novel mechanism for governing biofilm formation in Escherichia coli and Pseudomonas aeruginosa. Nitrite transporter is required for biofilm formation via regulation of NO levels and subsequent c-di-GMP production. Additionally, the nitrite transporter contributes more to P. aeruginosa virulence than quorum sensing. Thus, this study identifies nitrite transporters as new antibiofilm targets for future practical and therapeutic agent development.

## INTRODUCTION

Bacterial biofilms are well-organized surface-associated bacterial populations that subsist inside an extracellular matrix composed of extracellular polysaccharides, proteins, and extracellular DNA (eDNA) (1–3). The biofilm-forming capability of bacteria is linked to the antibiotic resistance and pathogenesis of numerous medically important bacterial strains (4–6). Common infections that are caused and sustained by bacterial biofilms include, but are not restricted to, lung infections of patients with cystic fibrosis (CF), ear infections, burn wound infections, catheter infections, chronic wound infections, tooth decay, and bacterial endocarditis (7). Biofilm-mediated infections prolong hospital stays, increase fatality, and consequently place a substantial financial burden on health care systems worldwide (8, 9). Thus, novel therapeutic strategies are needed to fight biofilm-mediated infections (10).

An example of a biofilm-forming multidrug-resistant bacterium is the opportunistic pathogen Pseudomonas aeruginosa, which was recently listed among the 12 antibiotic-resistant “priority pathogens” by the WHO (5, 7, 11). P. aeruginosa is fatal to CF patients, forming mucoid masses in lung tissue that lead to pneumonia; in addition, it causes severe infections in immunocompromised patients and is liable for the majority of nosocomial infections (12). Biofilm-grown P. aeruginosa persists despite frequent antibiotic treatment, causes reduced activation of complement, and shows less susceptibility to phagocytosis, indicating the role of biofilm formation on P. aeruginosa antibiotic resistance and virulence (4, 5).

P. aeruginosa biofilm formation is regulated by an intercellular chemical communication system, quorum sensing (QS) (13, 14). P. aeruginosa has three main QS systems: *las*, *rhl*, and *pqs.* Each QS system consists of autoinducer synthesis genes *lasI*, *rhlI*, and *pqsABCD*, as well as cognate regulatory genes *lasR*, *rhlR*, and *pqsR* (13). Bacteria within a biofilm display phenotypes distinct from those of planktonic cells, specifically those relating to growth and gene expression (4). QS systems are responsible for the expression of biofilm matrix genes and subsequent development of biofilm architecture (15). The intracellular second messenger cyclic di-GMP (c-di-GMP), synthesized by diguanylate cyclases (DGCs) and degraded by phosphodiesterases (PDEs) (16), can be regulated by QS systems because the activities of DGC- and c-di-GMP-specific PDE are reduced in the *lasR* and *rhlR* mutants, respectively (17). In addition, biofilm formation in P. aeruginosa is positively regulated by c-di-GMP (18, 19). c-di-GMP induces the transition from planktonic to biofilm lifestyles by downregulating motility-associated genes and upregulating exopolysaccharide- and biofilm maturation-associated genes (20). Conversely, low c-di-GMP levels induce biofilm dispersal by activating expression of motility mechanisms such as flagella and pili (21).

Recently, nitric oxide (NO) has also been reported to regulate biofilm dynamics in a wide variety of bacteria, including P. aeruginosa (22–24). P. aeruginosa produces NO via the denitrification pathway that reduces nitrate (NO_3_^−^) to dinitrogen (N_2_) via nitrite, nitric oxide (NO), and nitrous oxide (N_2_O) (25). Each step of the pathway is catalyzed by individual enzymes: nitrate reductase (NAR), nitrite reductase (NIR), nitric oxide reductase (NOR), and nitrous oxide reductase (NOS), respectively. Under anaerobic conditions, such as the ones encountered in the CF airway mucus and inside biofilms, P. aeruginosa can obtain sufficient energy through a denitrification pathway using nitrate (NO_3_^−^) and nitrite (NO_2_^−^) as final electron acceptors (26, 27). P. aeruginosa can obtain nitrate and nitrite from the host using nitrate and nitrite transporters (28, 29). The denitrification pathway is also active under aerobic conditions (23). Exogenous addition of nontoxic concentrations of NO (approximately nanomolar to micromolar) stimulates biofilm dispersal or inhibits biofilm formation in P. aeruginosa (30). The exogenous NO stimulates motility and biofilm dispersal in P. aeruginosa by enhancing PDE activity and subsequently decreasing c-di-GMP levels (23, 30, 31). Accordingly, P. aeruginosa biofilm dispersal requires expression of the nitrite reductase (NIR), an NO-generating enzyme in bacteria (30, 31). However, NIR and NO production are also reported to be important for biofilm formation (28, 32), which seemingly conflicts with their role in biofilm dispersal (30, 31). Although the regulation of biofilm dispersal by NIR-derived NO is relatively well understood (22, 33), how NIR is involved in biofilm formation is not yet understood.

The discovery and development of agents that can restrict biofilm formation or even eradicate established biofilms by targeting the above-mentioned pathways are urgently needed. Such therapeutic entities would have profound effects on antibiotic resistance and pathogenesis management (4, 29).

**FIG 1:**
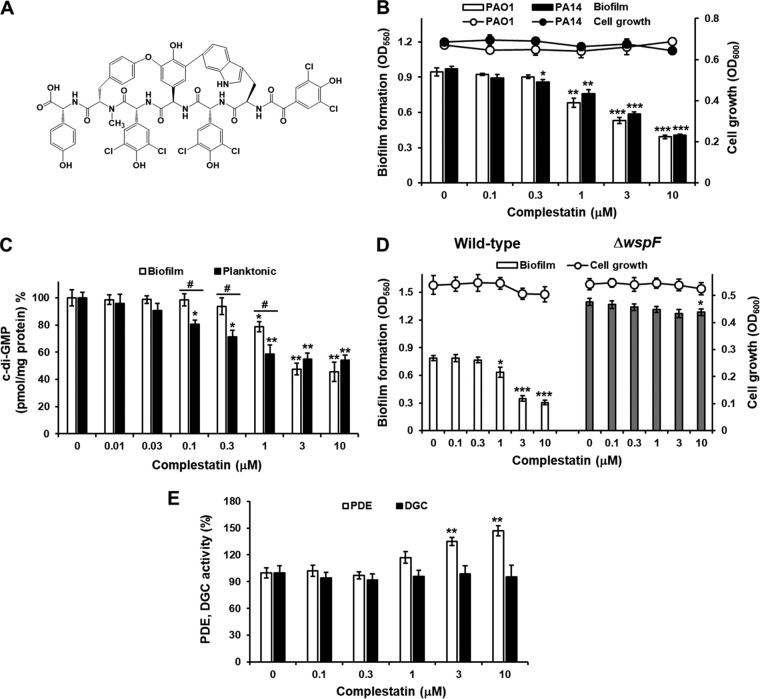
Complestatin inhibits P. aeruginosa biofilm formation by lowering cellular c-di-GMP levels via stimulation of PDE activity. (A) Chemical structure of complestatin. (B) Biofilm formation and cell viability in P. aeruginosa PAO1 and PA14 in the presence or absence of various concentrations of complestatin for 9 h, followed by the quantification of planktonic cells by measuring the optical density (OD) at 600 nm. Biofilm cells attached to the well surface were assayed using crystal violet staining. (C) Effects of complestatin on cellular c-di-GMP levels. Cellular cyclic-di-GMP (c-di-GMP) levels in biofilm and planktonic PA14 cultures grown in the presence of different complestatin concentrations for 9 h. After the biofilms were dissociated from the wells by gentle sonication, cellular c-di-GMP was extracted from the biofilm and planktonic cells, measured, and normalized to total protein. (D) Comparison of biofilm formation by the P. aeruginosa *wspF* mutant versus that by wild-type PA14 (pUCP18) containing only a vector cultured in the presence of different concentrations of complestatin for 9 h. White and gray bars represent the wild type and the *wspF* mutant biofilms, respectively. (E) PDE and DGC activities in PA14 cells cultured with different concentrations of complestatin for 6 h. Three independent experiments were performed, and the means ± standard deviation (SD) values are displayed as bars. *, *P* < 0.01; **, *P* < 0.001; ***, *P* < 0.0001 compared to untreated cells. #, *P* < 0.01.

Here, while screening a microbial metabolite library for a P. aeruginosa biofilm formation inhibitor, we identified complestatin (Fig. 1A), a structural analog of vancomycin, derived from a fermentation culture of Streptomyces chartreusis AN1542 (34). We then investigated the *in vitro* and *in vivo* effects of complestatin on inhibition of P. aeruginosa biofilm formation and the molecular mechanisms underlying this inhibition. Using P. aeruginosa and Escherichia coli *in vitro* biofilm formation systems and an E. coli mutant library, we found that complestatin enhances endogenous NO production to high levels via overactivation of NIR expression. Specifically, we identified the NasA nitrite transporter in P. aeruginosa as the complestatin target. We then demonstrated that the nitrite transporter partially suppressed NIR expression to produce low levels of NO necessary for biofilm formation. This can explain how NIR is involved in biofilm formation. Our findings also demonstrate that nitrite transporters are a new antibiofilm target.

## RESULTS

### Complestatin inhibits P. aeruginosa biofilm formation but not planktonic growth.

Screening of 5,839 microbial fermentation extracts for a P. aeruginosa PA14 biofilm formation inhibitor led to the selection of *S. chartreusis* AN1542. Bioactivity-guided fractionation of the mycelium of this strain identified complestatin that was previously isolated by our group (Fig. 1A) (35). Biofilm formation was significantly reduced by treatment with 1, 3, and 10 μM complestatin in a concentration-dependent manner in P. aeruginosa strains PAO1 and PA14 (Fig. 1B), whereas planktonic cell growth was not affected. The inhibitory effect on biofilm formation was not a result of a reduction in total cell numbers or cell viability as confirmed by optical density and viable cell growth assays (see Fig. S1A and B in the supplemental material). Conversely, vancomycin, a structural analog of complestatin, significantly induced biofilm formation at high concentrations (10 to 30 μM), whereas it had no effect on biofilm formation at lower concentrations (Fig. S1C). In addition, vancomycin inhibited planktonic cell growth at high concentrations (10 to 30 μM) (Fig. S1C).

10.1128/mBio.00878-20.1 FIG S1 Complestatin inhibits biofilm formation without affecting cell viability, but vancomycin induces biofilm formation in P. aeruginosa. (A) Effects of complestatin on bacterial growth. P. aeruginosa PA14 biofilms were formed in the presence of various concentrations of complestatin for 9 h, followed by the measurement of planktonic cells at 600 nm. After the biofilms were dissociated from the wells by gentle sonication and planktonic cells were harvested by centrifugation, the numbers of viable cells in the planktonic and biofilms were counted in each culture. (B) Growth-dependent effects of complestatin on biofilm formation and cell growth. Biofilms of PA14 were formed in medium containing different concentrations of complestatin for different times, followed by the measurement of planktonic cells at 600 nm. Three independent experiments were performed, and the means ± SD values are displayed as bars. *, *P* < 0.01; **, *P* < 0.001 versus untreated cells. (C) Effects of vancomycin on biofilm formation and cell growth of P. aeruginosa PA14. Two independent experiments were performed in triplicates, and the means ± SD values are displayed as bars. *, *P* < 0.01; **, *P* < 0.0001 versus untreated cells. Download FIG S1, TIF file, 0.1 MB. Copyright © 2020 Park et al. 2020 Park et al. This content is distributed under the terms of the Creative Commons Attribution 4.0 International license.

The effect of complestatin treatment (10 μM) on biofilm formation was further explored by confocal laser scanning microscopy (CLSM) using a BacLight Live/Dead Viability kit to stain the cells (Fig. S2A). The depth of complestatin-treated biofilms was approximately 40% less than that of untreated biofilms (Fig. S2B). The cell viable fluorescence intensity of complestatin treated P. aeruginosa PA14 biofilms was also reduced by 89.2% compared to that of untreated biofilms (Fig. S2C). Green fluorescence was not observed from dead bacteria.

10.1128/mBio.00878-20.2 FIG S2 Confocal laser scanning microscope analyses of P. aeruginosa biofilms treated with complestatin show biofilm inhibition, which is further confirmed by inhibition of extracellular polymer substance (EPS) production. (A to C) Confocal laser scanning microscope analyses of P. aeruginosa biofilms treated with complestatin. PA14 cells were grown on glass coverslips in a 24-well plate for 6 h in the medium containing 0 μM or 10 μM complestatin. (A) Biofilms were stained with the BacLight Live/Dead Viability kit. Cells that stained green were viable. The experiments were performed twice, and representative images are shown. (B) Biofilm thickness and quantification of green fluorescence intensities of two biofilms. Data represent the averages of image stacks collected from five randomly selected areas. *, *P* < 0.0001 versus biofilm from untreated PA14 cells. (C) The green fluorescence intensities in each of the sliced focal planes taken at 1.5-μm intervals are plotted as a function of biofilm height and compared between two biofilms. (D to F) Effects of complestatin on EPS production in biofilm formation of P. aeruginosa. P. aeruginosa PA14 biofilms formed in the presence of different concentrations of complestatin for 9 h. Three extracellular polymer substances, protein (D), polysaccharides (E), and extracellular DNA (eDNA) (F), were extracted and assessed from the biofilms. Planktonic cell densities were measured at 600 nm. Three independent experiments were performed in triplicates, and the means ± standard deviation (SD) values are displayed as bars. *, *P* < 0.01; **, *P* < 0.001 versus untreated cells. Download FIG S2, TIF file, 0.2 MB. Copyright © 2020 Park et al. 2020 Park et al. This content is distributed under the terms of the Creative Commons Attribution 4.0 International license.

To further confirm the biofilm inhibitory activity of complestatin, we quantified extracellular polymer substance (EPS) components in P. aeruginosa PA14 biofilms treated with or without complestatin. Complestatin (1 to 10 μM) significantly reduced the amounts of extracellular polysaccharides, proteins, and eDNA in P. aeruginosa biofilms compared to those in an untreated control (Fig. S2D to F). Overall, these results indicate that in contrast to vancomycin, complestatin inhibits P. aeruginosa biofilm formation without suppressing cell growth.

### Complestatin treatment decreases c-di-GMP levels.

To identify the mechanism by which complestatin inhibits biofilm formation, we measured the production of QS molecules [*N*-(3-oxododecanoyl)-l-homoserine lactone, *N*-butanoyl homoserine lactone, and 2-heptyl-3-hydroxy-4(1H) quinolone] and c-di-GMP in complestatin-treated P. aeruginosa PA14 biofilm cells. Treatment with furanone C-30 (FC), a well-known QS inhibitor, resulted in reductions in the levels of QS molecules and pyocyanin, a QS-related virulence factor, whereas treatment with complestatin (10 μM) had no effect (Fig. S3A to D). In contrast, c-di-GMP levels were significantly lower in PAO1 and PA14 biofilm cells treated with 1 to 10 μM complestatin than in untreated control cells (Fig. S3E). The effect was observed at lower complestatin concentrations in planktonic cells (0.1 and 0.3 μM) than that in biofilm cells (≥1 μM) (Fig. 1C). These results suggested that complestatin inhibits biofilm formation in P. aeruginosa by interfering with c-di-GMP formation.

10.1128/mBio.00878-20.3 FIG S3 Complestatin does not affect production of QS signaling molecules and pyocyanin, but inhibits intracellular c-di-GMP production in P. aeruginosa. (A to D) Effects of complestatin on production of QS signaling molecules and pyocyanin in P. aeruginosa PA14. P. aeruginosa was cultured in LB medium containing different complestatin or furanone C-30 (FC) concentrations for 24 h. The three main QS molecules, *N*-(3-oxododecanoyl)-l-homoserine lactone (OdDHL), *N*-butanoyl homoserine lactone (BHL), and 2-heptyl-3-hydroxy-4(1H) quinolone (PQS), were extracted from the culture supernatants and quantitatively analyzed by LC-MS/MS. The experiment shown is representative of two independent experiments in triplicates, and the means ± SD values are displayed as bars. *, *P* < 0.01; **, *P* < 0.001; ***, *P* < 0.0001 versus DMSO treatment. (E) Cellular cyclic-di-GMP (c-di-GMP) levels in biofilm PAO1 and PA14 cultures grown in the presence of different complestatin concentrations for 9 h. After the biofilms were dissociated from the wells by gentle sonication, cellular c-di-GMP was extracted from the biofilm, measured, and normalized to total protein. Three independent experiments were performed, and the means ± standard deviation (SD) values are displayed as bars. *P < 0.01, **P < 0.001, and ***P < 0.0001 compared to untreated cells. Download FIG S3, TIF file, 0.1 MB. Copyright © 2020 Park et al. 2020 Park et al. This content is distributed under the terms of the Creative Commons Attribution 4.0 International license.

Next, to confirm whether complestatin exerted its antibiofilm activity via inhibition of c-di-GMP production, P. aeruginosa PA14 biofilm formation inhibition by complestatin treatment was assessed in a c-di-GMP-overproducing mutant strain that displays enhanced biofilm formation (Δ*wspF*) (36). In the wild-type strain P. aeruginosa PA14, 3 μM complestatin inhibited biofilm formation by approximately 55.6%, and 10 μM complestatin inhibited biofilm formation by approximately 61% compared to that of an untreated control (Fig. 1D). In contrast, in the *wspF* mutant, inhibition of biofilm formation was only observed at the highest complestatin concentration tested (10 μM), and it was much lower than in the wild-type strain (9.3%) (Fig. 1D). In contrast, as expected, no differences in biofilm inhibition between the Δ*wspF* and wild-type strains were observed when using FC (data not shown) (17).

Overall, these results indicated that complestatin inhibits biofilm formation by decreasing cellular c-di-GMP levels.

### Complestatin enhances PDE activity but not DGC activity.

As DGCs and PDEs are responsible for c-di-GMP biosynthesis and degradation, respectively (16, 37), we measured PDE and DGC activities in P. aeruginosa PA14 cells treated with and without complestatin to understand the mechanism by which complestatin decreased intracellular c-di-GMP levels. We observed significantly higher PDE activity in complestatin-treated cells (3 to 10 μM) than in untreated cells, whereas DGC activity was not changed with 0.1 to 10 μM (Fig. 1E). These results suggest that complestatin decreases c-di-GMP levels by stimulating PDE activity and, hence, c-di-GMP degradation.

### NO is required for complestatin-mediated biofilm inhibition.

**FIG 2:**
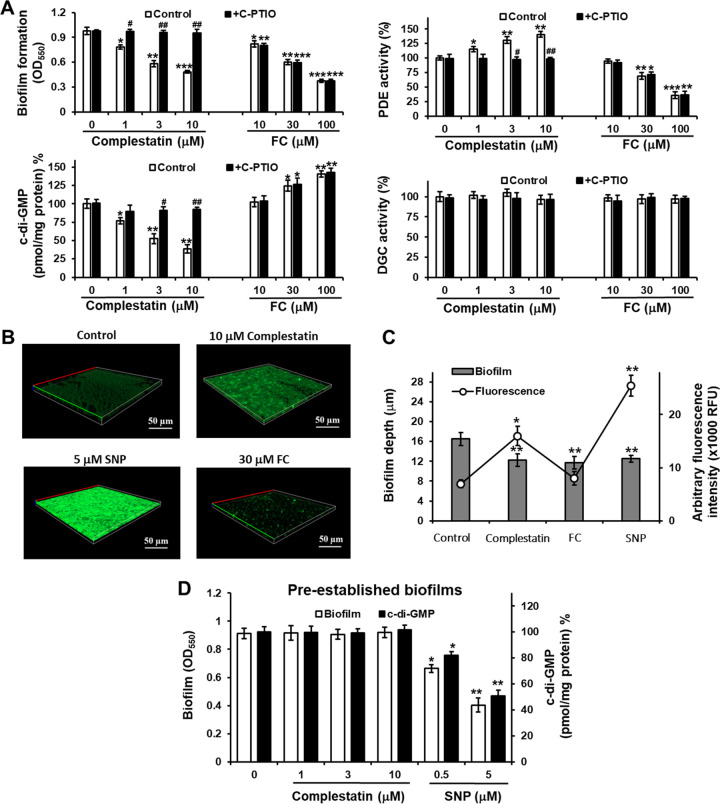
Complestatin-induced biofilm formation inhibition, reduction in c-di-GMP levels, and PDE activity stimulation are associated with increased NO levels and blocked by the NO scavenger C-PTIO. (A) Biofilm formation, c-di-GMP levels, and PDE and DGC activities in P. aeruginosa PA14 cultured in the presence of various concentrations of complestatin or FC and in the absence or presence of C-PTIO. Three independent experiments were performed, and the means ± standard deviation (SD) values are displayed as bars. *, *P* < 0.01; **, *P* < 0.001; ***, *P* < 0.0001 compared to untreated cells. #, *P* < 0.001; ##, *P* < 0.0001 compared to non-C-PTIO-treated cells. (B) Confocal microscopy images of complestatin-treated P. aeruginosa PA14 biofilms stained with the fluorescent NO probe DAF-2. The PA14 strain was grown on glass coverslips in a 24-well plate for 6 h in medium containing 0 μM complestatin, 10 μM complestatin, 5 μM SNP as a positive control, or 30 μM FC as a negative control. DAF-2 green fluorescence intensity indicates intracellular NO levels. The experiments were performed twice, and representative images are shown. (C) Biofilm thickness and quantification of DAF-2 green fluorescence. Data represent the averages derived from image stacks collected from five randomly selected areas. Two independent experiments were performed, and the means ± SD values are displayed as bars. *, *P* < 0.001; **, *P* < 0.000 compared to untreated cells. (D) Effects of complestatin on biofilms and c-di-GMP levels of preestablished biofilms. P. aeruginosa PA14 biofilms were preformed for 9 h and then treated with various concentrations of complestatin or SNP as a positive control for 12 h. Three independent experiments were performed, and the means ± SD values are displayed as bars. *, *P* < 0.001; **, *P* < 0.0001 compared to untreated cells.

Since NO stimulates biofilm dispersal (24) by stimulating PDE activity and subsequently decreasing c-di-GMP levels (31), we next tested whether the PDE-stimulating property of complestatin is NO dependent. To this end, we examined the effects of complestatin on biofilm formation, c-di-GMP levels, and PDE activity in P. aeruginosa in the presence of an NO scavenger, 2-(4-carboxyphenyl)-4,4,5,5-tetramethylimidazoline-1-oxyl-3-oxide (C-PTIO). We found that C-PTIO completely reversed the inhibition of biofilm formation, the decrease in c-di-GMP levels, and the stimulation of PDE activity caused by complestatin in P. aeruginosa, whereas it had no effect on DGC activity (Fig. 2A). In contrast, the NO scavenger did not reverse the effects of FC treatment on biofilm formation, c-di-GMP levels, or PDE activity (Fig. 2A).

To further confirm the role of NO in complestatin inhibition of biofilm formation, we next measured NO levels in biofilm cells of P. aeruginosa after complestatin treatment using CLSM intrabiofilm and the fluorescent NO probe 4,5-diaminofluorescein diacetate (DAF-2DA). NO levels were significantly higher in complestatin-treated P. aeruginosa cells than in untreated cells (Fig. 2B and C). Similarly, treatment with sodium nitroprusside (SNP; 5 μM), an NO donor that served as a positive control, resulted in higher NO levels and biofilm formation inhibition, whereas treatment with FC did not affect NO production but inhibited biofilm formation as expected (Fig. 2B and C).

Since high levels of exogenous NO (approximately nanomolar to micromolar) inhibits biofilm formation as well as disperses biofilms in P. aeruginosa (30), we tested the effect of complestatin on preestablished biofilms. Interestingly, complestatin did not affect dispersal and c-di-GMP levels of preestablished biofilms, whereas 5 μM SNP as a positive control did, as reported (30) (Fig. 2D).

Overall, these results indicated that complestatin elevates NO production in P. aeruginosa, followed by stimulation of PDE activity, a decrease in c-di-GMP levels, and biofilm formation inhibition.

### Complestatin inhibits biofilm formation by targeting the nitrite transporter NasA in P. aeruginosa.

**FIG 3:**
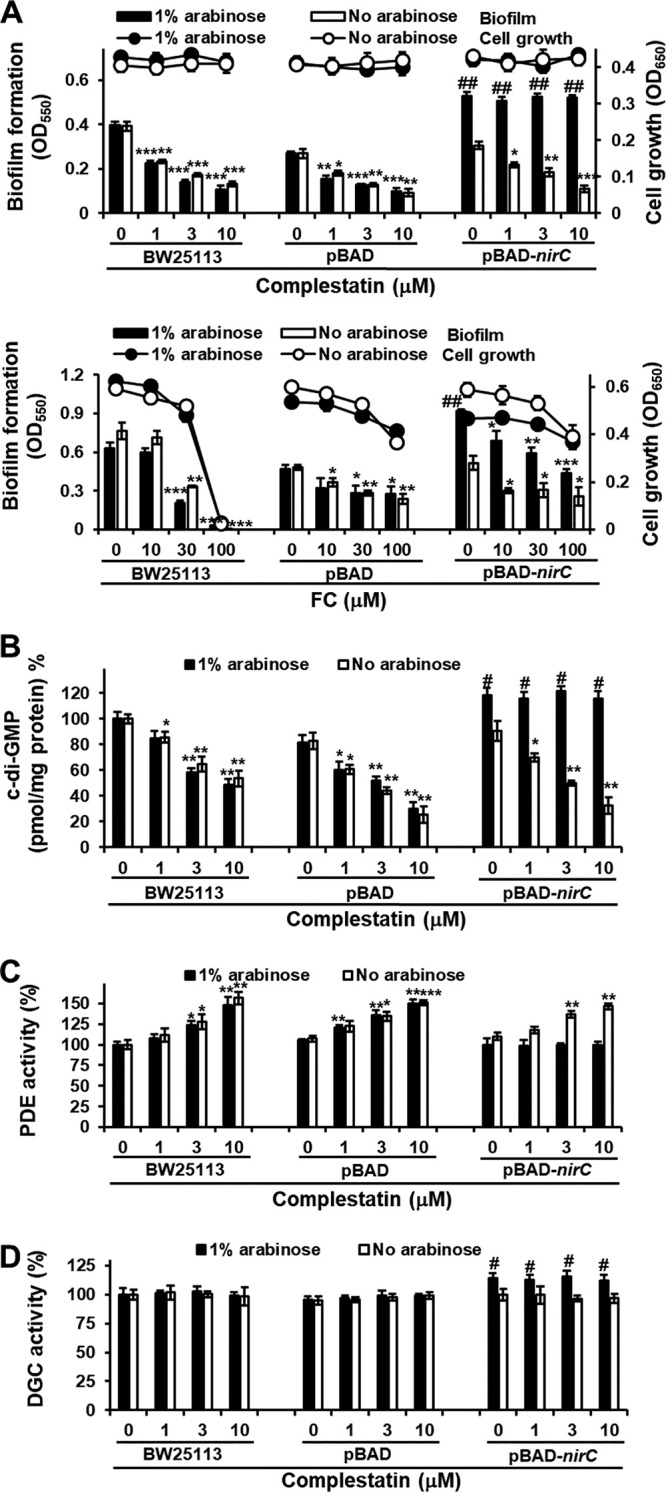
Overexpression of the nitrite transporter-encoding gene *nirC* in E. coli reverses the biofilm formation, c-di-GMP level, and PDE activity phenotypes induced by complestatin. Biofilm formation (A), intracellular c-di-GMP levels (B), and PDE (C) and DGC (D) activities in E. coli BW25113, E. coli BW25113 containing only a vector (pBAD), and a *nirC*-overexpressing E. coli BW25113 strain (pBAD-*nirC*) cultured in the presence of different concentrations of complestatin or FC and in the presence or absence of arabinose. Three independent experiments were performed, and the means ± standard deviation (SD) values are displayed as bars. *, *P* < 0.01; **, *P* < 0.001; ***, *P* < 0.0001 compared to untreated cells. #, *P* < 0.01; ##, *P* < 0.001 versus untreated BW25113.

We then used the knowledge of the mechanism involved in complestatin-mediated biofilm formation inhibition to identify the specific target protein of this compound. To this end, we used the extensive Keio E. coli gene knockout library, which consists of in-frame, single-gene deletion mutants for all nonessential genes in E. coli BW25113 (38). A knockout library with the same characteristics is not available in P. aeruginosa, and since these two bacteria are closely related, we decided to perform the screening in E. coli. To validate the use of E. coli as a surrogate of P. aeruginosa, we first checked that the mechanism of biofilm formation inhibition of complestatin in E. coli was the same as in P. aeruginosa. Complestatin inhibited biofilm formation, decreased c-di-GMP levels, and stimulated PDE activity in E. coli BW25113, the E. coli Keio knockout parent strain, and these effects were abrogated by treatment with C-PTIO (see Fig. S4). To identify the target protein of complestatin, we screened the E. coli Keio collection library for E. coli mutants that exhibited the same phenotype as complestatin-treated cells. Among the 3,801 E. coli Keio mutants tested, 11 showed 30% less biofilm formation than the wild-type BW25113, without cell growth being affected (see Fig. S5A). In 6 of the 11 mutants, biofilm formation was restored to wild-type levels in the presence of the NO scavenger C-PTIO (Fig. S5A), and these mutants were further screened for reduced c-di-GMP levels, increased PDE activity, and unchanged DGC activity compared to those of the wild type, which led to the selection of four mutants. The genes mutated in these four mutants (Δ*yhfS*, Δ*nirC*, Δ*ybfA*, and Δ*prfC*) were separately overexpressed in E. coli BW25113 using the plasmid pBAD with the arabinose-inducible promoter (see Table S1), and the effects of complestatin on biofilm formation and c-di-GMP levels were tested (Fig. S5B). In the presence of arabinose, complestatin failed to inhibit biofilm formation and c-di-GMP production and to enhance PDE activity, whereas it stimulated DGC activity only in a nitrite transporter (*nirC*)-overexpressing E. coli strain (Fig. S5B and Fig. 3A to D). In contrast, the QS inhibitor FC inhibited biofilm formation in the *nirC*-overexpressing E. coli strain regardless of the presence of arabinose, as expected (Fig. 3A).

10.1128/mBio.00878-20.4 FIG S4 Complestatin inhibits biofilm formation and intracellular c-di-GMP production and increases PDE activity in E. coli BW25113, and the effects are blocked by the NO scavenger C-PTIO. (A) Effects of complestatin on biofilm formation in the absence or presence of C-PTIO in E. coli BW25113. E. coli BW25113 biofilms were formed with various concentrations of complestatin in the absence or presence of C-PTIO for 18 h, followed by the measurement of planktonic cells at 600 nm. The biofilm cells attached to the well surface were assayed using crystal violet staining. (B) Effects of complestatin on cellular c-di-GMP. Cellular c-di-GMP levels in biofilm cultures of E. coli BW25113 grown with different complestatin concentrations in the absence or presence of C-PTIO for 18 h. After the biofilms were dissociated from the wells by gentle sonication, cellular c-di-GMP was extracted from the biofilm cells, measured, and normalized to the total proteins. Effects of complestatin on PDE (C) and DGC (D) activity. PDE and DGC activity in E. coli BW25113 cultured with different concentrations of complestatin in the absence or presence of C-PTIO for 24 h. Three independent experiments were performed in triplicates, and the means ± SD values are displayed as bars. *, *P* < 0.01; **, *P* < 0.001; ***, *P* < 0.0001 versus untreated cells. Download FIG S4, TIF file, 0.1 MB. Copyright © 2020 Park et al. 2020 Park et al. This content is distributed under the terms of the Creative Commons Attribution 4.0 International license.

10.1128/mBio.00878-20.5 FIG S5 Genome-wide screening of the Keio collection and overexpression assay result in selection of NirC as a target of complestatin. (A) Genome-wide screening of the Keio collection. Biofilms of Keio mutants representing 3,801 genes were assayed in 96-well plates. The 11 primary hits showing at least 30% less biofilm than the Keio parent strain E. coli BW25113 were reassayed in the presence of C-PTIO, where 6 hits showing enhanced biofilm production were selected as secondary hits. Three independent experiments were performed, and the means ± standard deviation (SD) values are displayed as bars. *P < 0.01; **P < 0.001. (B) Overexpression assay. Biofilm formation and intracellular c-di-GMP levels in E. coli BW25113, E. coli BW25113 containing only a vector (pBAD), and overexpression E. coli BW25113 strains (pBAD-*nirC*, pBAD-*yhfS*, pBAD-*ybfA*, and pBAD-*prfC*) cultured in the presence of different concentrations of complestatin and in the presence or absence of arabinose. Three independent experiments were performed, and the means ± standard deviation (SD) values are displayed as bars. *, *P* < 0.01; **, *P* < 0.001; ***, *P* < 0.0001 compared to untreated cells; #, *P* < 0.01; ##, *P* < 0.001; ###, *P* < 0.0001 versus untreated BW25113. Download FIG S5, TIF file, 0.2 MB. Copyright © 2020 Park et al. 2020 Park et al. This content is distributed under the terms of the Creative Commons Attribution 4.0 International license.

10.1128/mBio.00878-20.10 TABLE S1 Genes and primers used for construction of overexpression E. coli strains. Download Table S1, PDF file, 0.1 MB. Copyright © 2020 Park et al. 2020 Park et al. This content is distributed under the terms of the Creative Commons Attribution 4.0 International license.

**FIG 4:**
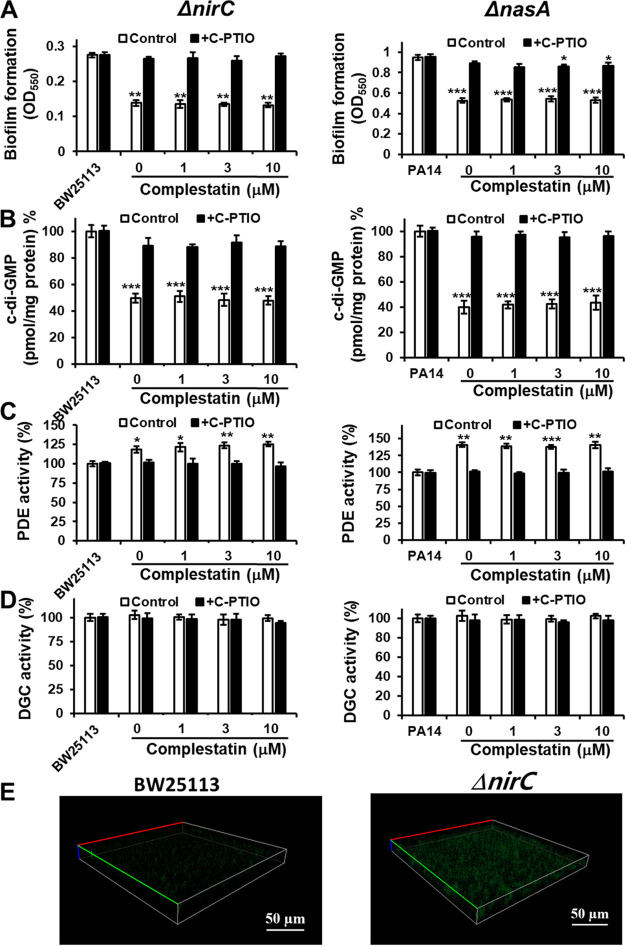
Deletion of the nitrite transporter-encoding genes in E. coli (*nirC*) and P. aeruginosa (*nasA*) supersedes the biofilm formation, c-di-GMP level, and PDE activity phenotypes induced by complestatin, and these effects are blocked by the NO scavenger C-PTIO. Biofilm formation (A), intracellular c-di-GMP levels (B), and PDE (C) and DGC (D) activities in an E. coli nitrite transporter mutant (Δ*nirC*) and a P. aeruginosa nitrite transporter mutant (Δ*nasA*) cultured in the presence of different complestatin or FC concentrations and in the absence or presence of C-PTIO. Three independent experiments were performed, and the means ± standard deviation (SD) values are displayed as bars. *, *P* < 0.01; **, *P* < 0.001; ***, *P* < 0.0001 compared to BW25113. (E). Intracellular NO increases in E. coli nitrite transporter mutant (Δ*nirC*). Confocal microscopy images of biofilms of wild-type E. coli BW25113 and E. coli *nirC* mutant stained with a fluorescent NO probe, DAF-2. DAF-2 green fluorescence indicated increased intracellular NO.

To further confirm the role of the nitrite transporter in complestatin-mediated inhibition of biofilm formation, we tested nitrite transporter mutants in E. coli BW25113 and P. aeruginosa PA14 (Δ*nirC* and Δ*nasA*, respectively). As expected, these mutants presented reduced biofilm formation, decreased c-di-GMP levels, and enhanced PDE activity even in the presence of complestatin, and these effects were reversed by C-PTIO treatment, whereas DGC activity was not affected under any of the conditions tested (Fig. 4A to D). Additionally, elevated NO production in the *nirC* mutant was confirmed by NO detection using CLSM (Fig. 4E). These results indicated that complestatin inhibits biofilm formation via NO production by targeting the nitrite transporters NirC and NasA in E. coli and P. aeruginosa, respectively.

### Nitrite transporters repress nitrite reductase transcription.

**FIG 5:**
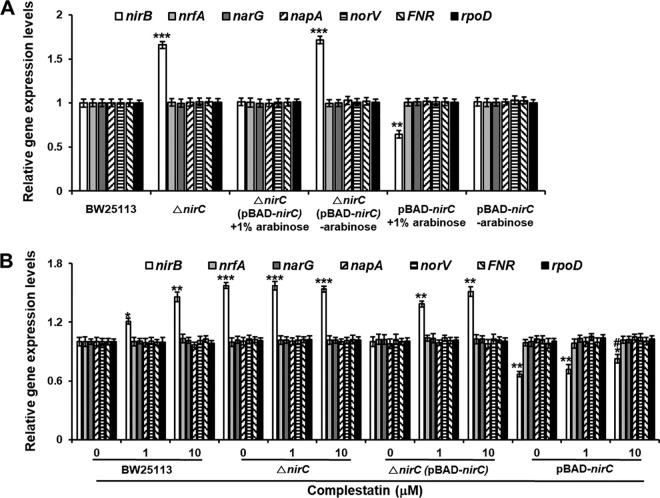
Expression of the nitrite reductase (NIR)-encoding gene *nirB* but not other NIR-encoding genes is elevated in the E. coli nitrite transporter mutant as well as in the presence of complestatin and suppressed in the nitrite transporter-overexpressing strain. (A) Expression of the NIR-encoding genes and related genes in wild-type E. coli BW25113, the E. coli nitrite transporter mutant Δ*nirC*, and the nitrite transporter-overexpressing strain pBAD-*nirC* as assessed by RT-qPCR. (B) Effects of complestatin on the expression of the NIR-encoding genes and related genes in BW25113, the Δ*nirC* strain, the Δ*nirC* strain complemented with *nirC* [Δ*nirC* (*pBAD-nir*)], and the pBAD-*nirC* strain as assessed by RT-qPCR. The experiment shown is representative of three independent experiments performed in triplicates, and the means ± SD values are displayed as bars. *, *P* < 0.01; **, *P* < 0.001; ***, *P* < 0.0001 versus dimethyl sulfoxide (DMSO) treatment. #, *P* < 0.01 versus untreated pBAB-*nirC*.

Next, to elucidate how complestatin ultimately inhibits biofilm formation, we investigated how the nitrite transporter inhibition by complestatin caused increased NO production. In bacteria, including E. coli and P. aeruginosa, NO is synthesized from nitrite by NIR. Thus, we hypothesized that the nitrite transporter represses transcription of the gene encoding NIR. To test this hypothesis, we analyzed the mRNA levels of the genes encoding NIRs in the E. coli nitrite transporter mutant Δ*nirC*, the Δ*nirC* mutant complemented with *nirC*, and a *nirC*-overexpressing strain by real-time quantitative PCR (RT-qPCR). In E. coli, there are two NIRs, a cytoplasmic NADH-dependent NIR (encoded by *nirBD*) and a periplasmic cytochrome *c*-dependent NIR (encoded by *nrfABCDEFG*), both of which can reduce nitrite to NO (39, 40). We analyzed the mRNA levels of the first gene in each NIR operon. mRNA levels of the cytoplasmic NADH-dependent NIR (*nirB*) dramatically increased in the *nirC* mutant compared to those in the wild-type BW25113, whereas those of the periplasmic formate-dependent NIR (*nrfA*) were not changed (Fig. 5A). The increased *nirB* levels in the *nirC* mutant returned to normal in the *nirC* complemented strain (Fig. 5A). The decrease in *nirB* transcription in response to *nirC* overexpression confirmed that NirC suppressed *nirB* transcription and, importantly, also indicated that NirC partially suppressed *nirB* transcription in the wild-type E. coli (Fig. 5A), which is consistent with NO detection in wild-type E. coli BW25113 biofilm cells (Fig. 4E). Additionally, NIR suppression by the nitrite transporter was demonstrated in P. aeruginosa. P. aeruginosa has only one type of NIR (encoded by *nirS*), which is periplasmic. The mRNA levels of *nirS* were increased 1.5-fold in the nitrite transporter Δ*nasA* mutant (see Fig. S6A). These results indicated that the nitrite transporter partially suppressed the transcription of NIRs in both E. coli and P. aeruginosa.

10.1128/mBio.00878-20.6 FIG S6 Expression of the nitrite reductase (NIR)-encoding gene *nirS* is elevated in a nitrite transporter mutant (Δ*nasA*) of P. aeruginosa and enhanced by complestatin in P. aeruginosa. (A) Expression of *nirS* but not other NIR-encoding genes is elevated in a Δ*nasA* mutant of P. aeruginosa and is more severe in the presence of nitrate. Expression of *nirS* and related genes in wild-type PA14, the Δ*nasA* mutant, the Δ*nirS* mutant, the Δ*lasR* mutant, and the Δ*rhlR* mutant cultured in presence or absence of nitrate as assessed by RT-qPCR. The experiment shown is representative of three independent experiments in triplicates, and the means ± SD values are displayed as bars. *, *P* < 0.01; **, *P* < 0.001; #, *P* < 0.0001 versus PA14. ***, *P* < 0.01 versus no nitrate treatment. (B to D) Complestatin enhances expression of *nirS* in P. aeruginosa PAO1 and PA14, and deletion of *nasA* supersedes expression of *nirS* induced by complestatin. Expression of *nirS* and related genes in P. aeruginosa PAO1 (B), PA14 (C), and the Δ*nasA* mutant (D) cultured with complestatin as assessed by RT-qPCR. The experiment shown is representative of three independent experiments in triplicates, and the means ± SD values are displayed in each bar. *, *P* < 0.01; **, *P* < 0.001; ***P < 0.0001 versus DMSO treatment. Download FIG S6, TIF file, 0.2 MB. Copyright © 2020 Park et al. 2020 Park et al. This content is distributed under the terms of the Creative Commons Attribution 4.0 International license.

Next, we tested whether complestatin induced the transcription of NIR via inhibition of a nitrite transporter. In E. coli, complestatin treatment resulted in a dose-dependent increase in *nirB* mRNA levels in the wild-type BW25113 but not in the Δ*nirC* mutant, whereas the effect of complestatin on *nirB* mRNA levels was restored in the Δ*nirC* strain complemented with *nirC* (Fig. 5B). Moreover, an increase in *nirS* transcription was observed in both PAO1 and PA14 treated with complestatin but not in the nitrite transporter mutant (Δ*nasA*) (Fig. S6B to D). These results indicated that complestatin restored the transcription of NIR via nitrite transporter inhibition in both E. coli and P. aeruginosa.

### Nitrite reductase, partially suppressed by nitrite transporter, produces NO that activates DGC to produce c-di-GMP.

**FIG 6:**
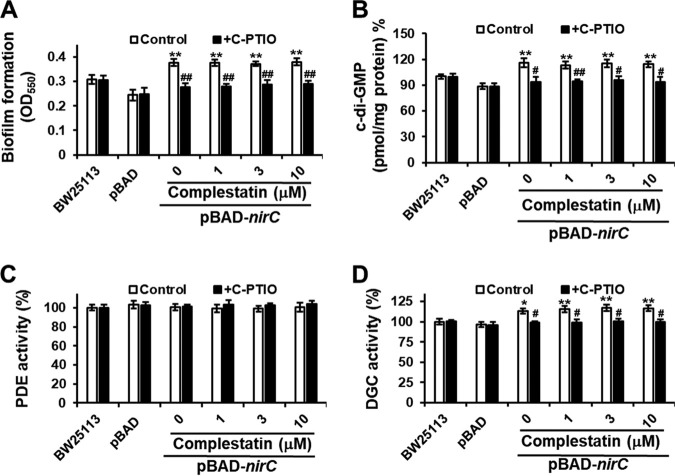
Overexpression of the nitrite transporter-encoding gene *nirC* in E. coli induces biofilm formation enhancement, increases in c-di-GMP levels, and DGC activity stimulation, and these effects are blocked by the NO scavenger C-PTIO. Biofilms (A), c-di-GMP levels (B), and PDE (C) and DGC (D) activities in E. coli BW25113, E. coli BW25113 containing only a vector (pBAD), and a *nirC*-overexpressing E. coli BW25113 strain (pBAD-*nirC*) cultured with complestatin and 1% arabinose in the absence or presence of C-PTIO. Three independent experiments were performed, and the means ± standard deviation (SD) values are displayed as bars. *, *P* < 0.01; **, *P* < 0.001 compared to pBAD cells. #, *P* < 0.01; ##, *P* < 0.001 compared to C-PTIO-untreated cells.

Because the nitrite transporter partially suppressed the transcription of NIRs in the wild type and, as shown in Fig. 3A and B, nitrite transporter overexpression enhanced biofilm formation and c-di-GMP production, we hypothesized that NO, produced by the partially suppressed NIR, stimulates c-di-GMP production and biofilm formation. Indeed, in E. coli, *nirC* overexpression enhanced c-di-GMP production and biofilm formation, and these effects were revered by C-PTIO treatment (Fig. 6A and B). These results indicated that NIR, partially suppressed by NirC, produced endogenous NO that caused c-di-GMP production and subsequent biofilm formation.

Next, given that at low concentrations, endothelial NO synthase-derived NO binds to soluble guanylyl cyclase (sGC) to produce cyclic-GMP (c-GMP) in mammals (41), we hypothesized that a limited amount of NO produced by a partially suppressed NIR activates DGCs to produce c-di-GMP for biofilm formation. Indeed, *nirC* overexpression enhanced DGC activity but did not affect PDE activity, which was prevented by C-PTIO treatment (Fig. 6C and D). This result indicated that NIR, partially suppressed by NirC, produced endogenous NO that in turn activated DGCs.

Thus, taken together, these results indicate that NO derived from nitrite transporter-regulated NIR activated DGCs to produce c-di-GMP for biofilm formation.

### Nitrite transporter mutant shows increased survival of Caenorhabditis elegans compared to that of wild-type PA14 and the QS mutants.

**FIG 7:**
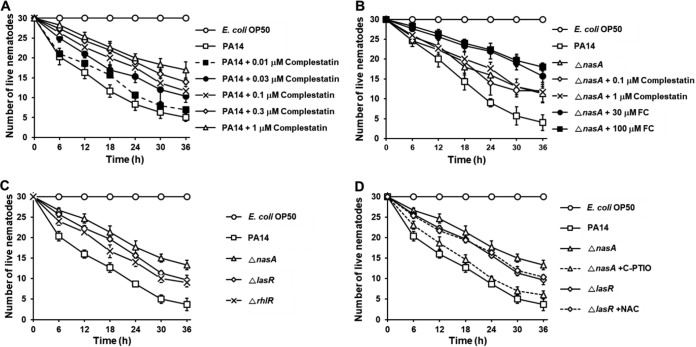
Nitrite transporter contributes more to P. aeruginosa virulence than QS. (A) Complestatin suppresses P. aeruginosa virulence toward C. elegans. C. elegans was applied to E. coli OP50 or PA14 lawns on plates containing different concentrations of complestatin. Live nematodes were counted every 6 h for 36 h. Three independent experiments were performed in triplicates, and the means ± SD values are displayed in each graph. The presence of complestatin (0.1 to 1 μM) significantly protected C. elegans from being killed (*P* < 0.001). (B) The PA14 nitrite transporter mutant Δ*nasA* has reduced virulence toward C. elegans. C. elegans was applied to E. coli OP50, PA14, or the *nasA* mutant lawns on plates containing different concentrations of complestatin or furanone C-30 (FC). The *nasA* mutant was significantly less virulent than wild-type PA14 (*P* < 0.01). The presence of FC (30 to 100 μM) significantly protected C. elegans from Δ*nasA*-mediated killing (*P* < 0.01). (C) The *nasA* mutant has less virulence toward C. elegans than the QS mutants. C. elegans was applied to E. coli OP50, PA14, the *nasA* mutant, or QS mutant (Δ*lasR* and Δ*rhlR*) lawns on plates. The *nasA* mutant was less virulent than the *lasR* and *rhlR* mutants (*P* < 0.01). (D) The virulence-reducing property of the *nasA* mutant is NO dependent. C. elegans was applied to E. coli OP50, PA14, the *nasA* mutant, or the *lasR* mutant lawns on plates in the absence or presence of the NO scavenger C-PTIO. Reduced virulence of the *nasA* mutant was recovered by C-PTIO (*P* < 0.001).

Biofilm extracellular matrix plays a role in P. aeruginosa virulence by improving microbial attachment for invasion and avoiding phagocytosis (4, 5). P. aeruginosa mediates pathogenesis via biofilm-mediated production of pyoverdine, a virulence factor, in a C. elegans animal model (42). Thus, we first checked if complestatin was able to attenuate P. aeruginosa-mediated pathogenesis *in vivo* using C. elegans, a well-established and practical model for studying P. aeruginosa virulence (43–46). C. elegans rapidly died when fed P. aeruginosa PA14, as evidenced by only 20% survival 30 h postinfection (Fig. 7A). Treatment with complestatin (0.01 to 1 μM) and P. aeruginosa at the same time resulted in a dose-dependent increase in nematode survival, up to 67% survival at the highest concentration tested (Fig. 7A). We then tested whether the protective effects of complestatin on killing of C. elegans by PA14 were also the result of inhibition of the nitrite transporter NasA by comparing the virulence of the *nasA* mutant with that of the wild-type PA14. Indeed, the *nasA* mutant showed increased survival of C. elegans compared to that of the wild-type PA14 (Fig. 7B). Complestatin treatment had no effect in C. elegans infected with the *nasA* mutant strain, as expected. In contrast, the QS inhibitor FC further reduced the virulence of the *nasA* mutant. These results confirmed that the nitrite transporter is the target of complestatin in C. elegans infection.

Next, we compared the virulence of the nitrite transporter mutant with that of the QS mutants. The *nasA* mutant exhibited increased survival of C. elegans compared to that with the *lasR* and *rhlR* mutants (Fig. 7C). The virulence-reducing property of the *nasA* mutant was reversed by C-PTIO treatment, whereas that of the QS mutants was not, suggesting that the *nasA* virulence *in vivo* was NO dependent similarly to that *in vitro* (Fig. 7D). These results indicated that NasA contributes more to P. aeruginosa virulence via NO production than QS.

Overall, these data suggest that NasA is a potential therapeutic target against P. aeruginosa infection *in vivo*.

### Inhibition of biofilm formation by targeting the nitrite transport is enhanced in the presence of nitrate.

Because complestatin produced NO via NIR activation of the denitrification pathway using nitrate/nitrite, complestatin is expected to increase NO production and consequently biofilm formation inhibition in the presence of nitrate. Indeed, complestatin increased NIR (*nirS*) induction and decreased biofilm in PA14 supplemented with 15 mM nitrate (see Fig. S7A). Complestatin inhibited biofilm formation three times more potently in nitrate-supplemented medium than in control medium, while the QS inhibitor FC did not display this discrepancy (Fig. S7B). Similarly, complestatin reduced cellular c-di-GMP levels with increased potency in the presence of nitrate (Fig. S7C). This finding was confirmed by comparing the nitrite transporter mutant to QS mutants. The increased NIR expression and reduced biofilm formation and c-di-GMP levels in the nitrite transporter mutant (Δ*nasA*) in P. aeruginosa were more severe in the presence of nitrate than in its absence, whereas they did not change in the QS mutants, i.e., *lasR* and *rhlR* mutants (Fig. S6A and S8). These results indicated that biofilm formation inhibition by targeting the nitrite transporter was potentiated in the presence of nitrate.

10.1128/mBio.00878-20.7 FIG S7 Expression of NIR, inhibition of biofilm formation, and reduction in c-di-GMP levels induced by complestatin are enhanced in the presence of nitrite. Expression of nitrite reductase gene (*nirS*) and related genes (A), biofilms (B), and c-di-GMP levels (C) in P. aeruginosa PA14 cultured with various concentrations of complestatin in absence or presence of nitrate. Three independent experiments were performed, and the means ± standard deviation (SD) values are displayed as bars. *, *P* < 0.01; **, *P* < 0.001; ***, *P* < 0.0001 compared to untreated cells. #, *P* < 0.01; ##, *P* < 0.001; ###, *P* < 0.0001. Download FIG S7, TIF file, 0.2 MB. Copyright © 2020 Park et al. 2020 Park et al. This content is distributed under the terms of the Creative Commons Attribution 4.0 International license.

10.1128/mBio.00878-20.8 FIG S8 Inhibition of biofilm formation and c-di-GMP levels induced by deletion of nitrite transporter are more severe in the presence of nitrate. Biofilms (A) and c-di-GMP levels (B) in the nitrite transporter mutant (Δ*nasA*) cultured in presence or absence of nitrate, compared with the wild-type PA14 and the QS mutants (Δ*lasR* and Δ*rhlR*). Three independent experiments were performed, and the means ± standard deviation (SD) values are displayed as bars. *, *P* < 0.01 compared to untreated cells. Download FIG S8, TIF file, 0.1 MB. Copyright © 2020 Park et al. 2020 Park et al. This content is distributed under the terms of the Creative Commons Attribution 4.0 International license.

### Combined inhibition of two different mechanisms, nitrite transport and QS, increases biofilm formation prevention.

**FIG 8:**
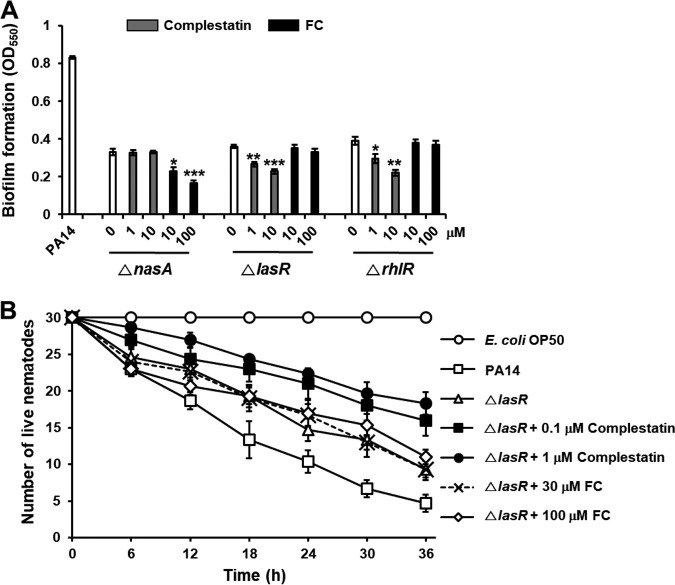
Combined inhibition of nitrite transporter and QS enhances biofilm formation inhibition and P. aeruginosa virulence prevention. (A) Effects of complestatin and furanone C-30 (FC) on biofilm formation in the nitrite transporter mutant (Δ*nasA*) and the QS mutants (Δ*lasR* and Δ*rhlR*) of P. aeruginosa. Biofilms in wild-type PA14, the Δ*nasA* mutant, the Δ*lasR* mutant, and the Δ*rhlR* mutant cultured with different concentrations of complestatin or FC. Three independent experiments were performed, and the means ± standard deviation (SD) values are displayed as bars. *, *P* < 0.01; **, *P* < 0.001; ***, *P* < 0.0001 compared to untreated cells. (B) Potentiation effects of complestatin on virulence of the Δ*lasR* mutant toward C. elegans. C. elegans was applied to lawns of E. coli OP50 or PA14 Δ*lasR* mutant on plates containing different concentrations of complestatin or FC. The live nematodes were counted every 6 h for 36 h. Two independent experiments were performed in triplicates, and the means ± SD values are displayed in each graph. Δ*lasR* mutant was significantly less virulent than PA14 (*P* < 0.001). The presence of complestatin (0.1 to 1 μM) significantly protected C. elegans from the Δ*lasR* mutant (*P* < 0.01).

Nitrite transporters enhanced biofilm formation via a completely different mechanism than the QS system in P. aeruginosa and E. coli. It was suggested that combined inhibition of the two mechanisms could prevent biofilm formation, because biofilms were not inhibited completely in either nitrite transporter or QS mutants. Indeed, FC, a QS inhibitor, inhibited the residual biofilm formation in the *nasA* mutant in a dose-dependent manner (49.5% more reduction by 100 μM FC) (Fig. 8A). Conversely, complestatin effectively eradicated the residual biofilms of the *lasR* and *rhlR* mutants (36.5% and 43.4% more reduction by 10 μM complestatin, respectively) (Fig. 8A). The profound combined effects were also verified in the C. elegans *in vivo* model (Fig. 6B and 8B). These results indicated that combined inhibition of nitrite transporter and QS more effectively inhibited biofilm formation and prevented P. aeruginosa virulence *in vivo.*

## DISCUSSION

**FIG 9:**
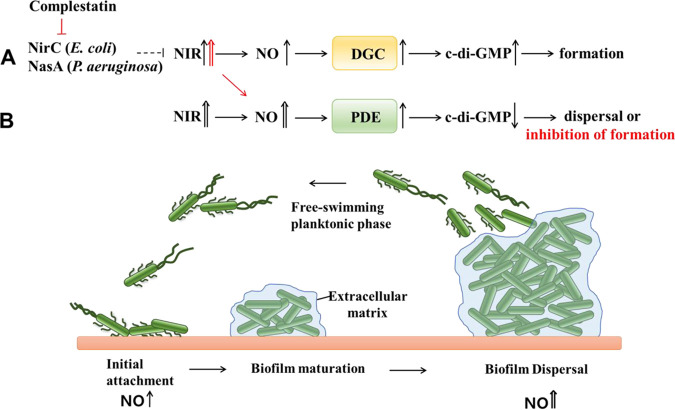
Nitrite reductase (NIR)-derived NO plays roles in both biofilm formation and dispersal in P. aeruginosa. (A) NO-mediated biofilm formation. This study suggested that NO-mediated biofilm formation occurred via the appropriate suppression of NIR by a nitrite transporter (NasA and NirC of P. aeruginosa and E. coli, respectively). The controlled NO production activated DGC but not PDE activity, which led to an increase in intracellular c-di-GMP levels and subsequent biofilm formation. (B) NO-mediated biofilm dispersal, which is relatively well understood and reviewed (23, 31). Complestatin inhibited biofilm formation by the following sequential processes: nitrite transporter inhibition, NIR desuppression, high NO production, PDE activity stimulation, and c-di-GMP level reduction.

NIR, which produces NO, is reported to be involved in both biofilm formation and dispersal, conflicting processes, in P. aeruginosa. The mechanism by which NIR regulates biofilm dispersal is relatively understood, but there are no reports about how NIR is involved in biofilm formation (23). In this study, we found that complestatin inhibited c-di-GMP production and biofilm formation by targeting the nitrite transporters in E. coli and P. aeruginosa. We then demonstrated that the nitrite transporter partially suppressed NIR, which produced the limited amount of NO to activate DGCs, not PDEs. The activated DGCs produce c-di-GMP for biofilm formation. These results provided a novel mechanism for the NO requirement in biofilm formation (Fig. 9A). We have further shown that nitrite transporter contributes to P. aeruginosa virulence *in vivo* more than the QS receptors, LasR and RhlR, and combined inhibition of nitrite transporter and the QS targets enhances both antibiofilm activity and antivirulence effects.

In other reports, preestablished biofilms were dispersed by NO via enhancement of PDE activity and subsequent reduction in c-di-GMP levels (31), and biofilm formation was inhibited by terrein, a QS inhibitor, via decreases in DGC activity and the subsequent reduction in c-di-GMP levels (17). In this study, complestatin inhibited biofilm formation at 1 to 10 μM without affecting cell viability by lowering cellular c-d-GMP levels in PAO1 and PA14 without affecting QS systems. Instead, complestatin enhanced c-di-GMP-specific PDE activity in PAO1 and PA14 without dispersing preestablished biofilms. To our knowledge, this is the first description of the inhibition of biofilm formation by a drug that enhances PDE activity and subsequently reduces c-di-GMP levels.

NO is an important factor regulating biofilm formation and mediating changes in the biofilm life cycle through c-di-GMP and/or QS in a wide variety of bacteria (23, 24). NIR, which produces NO, is reported to be involved in both biofilm formation and dispersal, conflicting processes, in P. aeruginosa. The mechanism by which NIR regulates biofilm dispersal is relatively understood, but there are no reports about how NIR is involved in biofilm formation (23). A P. aeruginosa NIR (*nirS*) mutant produces poorly dispersing biofilms and partially regains dispersal ability upon exogenous NO addition, indicating that endogenous NO produced by NIR is essential for P. aeruginosa biofilm dispersal (30, 31). On the other hand, a P. aeruginosa NIR (*nirS*) mutant failed to form biofilms under anaerobic conditions, indicating the requirement of NIR and NO production for biofilm formation (32). Additionally, biofilm formation and c-di-GMP levels were considerably reduced in a P. aeruginosa NIR (*nirS*) mutant under aerobic conditions (28). In this study, complestatin inhibited biofilm formation through the activation of NIR expression, and the nitrite transporter enhanced biofilm formation through the suppression of NIR expression in P. aeruginosa, which contrasts with the previous results (28, 32) that showed that NIR is necessary for biofilm formation under both anaerobic and aerobic conditions. However, interestingly, this study also showed that nitrite transporter overexpression greatly enhanced biofilm formation through increased suppression of NIR expression. These results strongly suggested that the nitrite transporter partially suppressed NIR expression in the wild type so that NIR was expressed to specific levels to produce particular low-level NO during biofilm formation, which might be a requirement for biofilm formation.

In this study, the relatively low levels of NO produced when NIR was suppressed by *nirC* overexpression led to increased biofilm formation and c-di-GMP, whereas the high levels of NO produced when NIR was desuppressed in the *nirC* mutant led to a reduction in biofilm formation and c-di-GMP. The phenotype that biofilm formation is inhibited by NO has been reported with high levels of exogenous NO (approximately nanomolar to micromolar) in P. aeruginosa (30) (Fig. 2C), but stimulation of biofilm formation by relatively low levels of exogenous NO has not yet been reported. Notably, it is reported that the high levels of exogenous NO not only inhibit biofilm formation but also disperse biofilms in P. aeruginosa (30). Interestingly, complestatin inhibited biofilm formation and c-di-GMP production via NasA inhibition, NIR desuppression, and subsequent high NO production but had no effects on dispersal and c-di-GMP levels of preestablished biofilms (Fig. 2D). These results suggested that the NasA gene is active in planktonic cells or early-stage biofilms, not matured biofilms (Fig. 9A and B).

Given that at low concentrations, endothelial NO synthase-derived NO binds to soluble guanylyl cyclase (sGC) to produce cyclic-GMP (c-GMP) in mammals (41), we hypothesized that a limited amount of NO produced by a partially suppressed NIR activates DGCs to produce c-di-GMP for biofilm formation (Fig. 9A), whereas a relatively large amount of NO produced under normal NIR activity conditions, which is somehow derepressed after biofilm maturation, activates PDEs to degrade c-di-GMP for biofilm dispersal or inhibition of biofilm formation (Fig. 9B). Here, we present evidence that the NO produced while NIR was suppressed by a nitrite transporter (NirC) activated DGCs in E. coli to produce c-di-GMP for biofilm formation. First, *nirC* overexpression enhanced DGC activity but did not affect PDE activity, which was prevented by C-PTIO treatment. This result indicated that NIR, partially suppressed by NirC, produced endogenous NO that in turn activated DGCs. Second, using a PA14 NIR mutant (Δ*nirS*), it was confirmed that NIR is necessary for biofilm formation in this system. Indeed, biofilm formation and c-di-GMP levels were dramatically reduced in the Δ*nirS* mutant (data not shown), indicating that NIR is essential for biofilm formation, which is consistent with the previous reports of Zhou et al. (28) and Yoon et al. (32). Thus, taken together, these results indicate that NO derived from nitrite transporter-regulated NIR activated DGCs to produce c-di-GMP for biofilm formation. Although the detailed mechanisms of NO-mediated DGC activation remain to be clarified, this result could explain the unanswered question of how NIR plays roles in both biofilm formation and dispersal (Fig. 9).

Nitrite is a central intermediate in the nitrogen metabolism of microorganisms and plants. The bacterial membrane nitrite transporter protein catalyzes nitrite uptake and export across the cytoplasmic membrane; this nitrite is subsequently reduced by cytoplasmic or periplasmic NIRs to produce NO or NH_4_^+^ for nitrogen metabolism and cytoplasmic detoxification (47, 48). Here, we present evidence supporting a critical role for nitrite transporters in biofilm formation in E. coli and P. aeruginosa. First, complestatin inhibited biofilm formation and potently prevented P. aeruginosa virulence in C. elegans, and the complestatin target was identified as a nitrite transporter through a genome-wide screen of the Keio collection, which was validated using a nitrite transporter-overexpressing strain. Second, E. coli and P. aeruginosa nitrite transporter mutants exhibited reduced biofilm formation and c-di-GMP levels, whereas *nirC* overexpression increased biofilm formation. Furthermore, it was demonstrated that the nitrite transporter partially suppressed NIR transcription, which caused the following sequential processes: low NO production, followed by DGC activation, c-di-GMP production, and, ultimately, biofilm formation stimulation.

P. aeruginosa produced NO by NIR of the denitrification pathway using nitrate/nitrite. Given the fact that complestatin and the nitrite transporter mutant produced NO via NIR activation, complestatin treatment and the nitrite transporter mutant are expected to increase NO production and consequently biofilm formation inhibition in the presence of nitrate. Indeed, complestatin more potently decreased biofilm and c-di-GMP levels in the presence of nitrate. The nitrite transporter mutant also presented greater reductions in biofilm formation and c-di-GMP levels in the presence of nitrate. Of note, NIR expression in the nitrite transporter mutant was elevated in the presence of nitrite, whereas that in the wild-type and QS mutants (*lasR* and *rhlR* mutants) was not. Considering that sufficient nitrite is present in CF airway surface liquid and sputum (27, 49), these data suggest that nitrite transporter inhibitors could have important clinical implications and advantages over QS inhibitors.

Denitrification genes such as *nar*, *nir*, *nor*, and *nos* are induced under anaerobic or low-oxygen conditions in the presence of nitrate or nitrite in P. aeruginosa (50, 51). Recently, it was reported that denitrification can also be activated under aerobic conditions in P. aeruginosa (23, 28). Expression of denitrification genes is tightly controlled by the arginine nitrate regulator (ANR) and dissimilative nitrate regulator (DNR) transcription factors. The master regulator ANR, a homologue of E. coli FNR (a well-characterized oxygen-sensing regulator), activates another CRP/FNR-related transcriptional DNR under anaerobic or low-oxygen conditions (51, 52), which in turn activates transcription of all denitrification genes (50). The two-component nitrate sensor-response regulator NarX/NarL in cooperation with ANR induces the expression of the genes *narK*, *dnr*, *nirQ*, and *nar* (51). Another anaerobic NIR regulator, NirQ, is predicted to be involved in fine-tuning the expression and activation of NIR and nitric oxide reductase (51). In E. coli, FNR activates transcription of the denitrification genes. In this study, among the denitrification genes, mainly NIR genes (*nirS*) were activated dose dependently in complestatin-treated PAO1 and PA14, although *narG* (plasmic nitrate reductase) was a somewhat activated in PA14 (see Fig. S6B and C in the supplemental material). Among transcriptional regulators, *nirQ* was somewhat activated in PAO1 and PA14 (Fig. S6B and C). However, given the fact that mainly NIR was activated, it was suggested that other regulation might be involved. In the *nasA* mutant of P. aeruginosa, transcription of mainly NIR was dramatically activated, although *narG* was somewhat activated in PA14, which is consistent with that in complestatin-treated cells (Fig. S6A). Additionally, in E. coli, transcription of only NIR was affected in complestatin-treated cells, the *nirC* mutant, and the *nirC*-overexpressing strain, whereas that of other genes and FNR were not affected (Fig. 5). These results indicate that the nitrite transporter suppressed transcription of NIRs, which could occur via an unknown mechanism. Also, the possibility of the nitrite transporter itself as a transcriptional factor cannot be excluded.

By investigating the mechanism of biofilm formation with complestatin, this study describes a novel mechanism governing biofilm formation in E. coli and P. aeruginosa. Nitrite transporter was identified as a protein target of complestatin. Nitrite transporter partially suppressed NIR in E. coli and P. aeruginosa. NO, produced by the partially suppressed NIR, activated DGCs, not PDEs, and subsequently produced c-di-GMP that is essential for biofilm formation. This finding indicates that the partial suppression of NIR by nitrite transporter is a prerequisite for biofilm formation in E. coli and P. aeruginosa, and NO plays roles in both biofilm formation and dispersal, conflicting processes, via differential regulation of NIR. Thus, this study provides nitrite transporters as new antibiofilm targets. In this respect, our findings provide new insight into how the biofilm life cycle is regulated through NO and how biofilm can be prevented.

## MATERIALS AND METHODS

See Text S1 in the supplemental material for additional details regarding the materials and methods.

10.1128/mBio.00878-20.9 TEXT S1 Supplemental materials and methods. Download Text S1, DOCX file, 0.1 MB. Copyright © 2020 Park et al. 2020 Park et al. This content is distributed under the terms of the Creative Commons Attribution 4.0 International license.

### Materials.

Complestatin was isolated from Streptomyces chartreusis AN1542 mycelia as stated in our previous study (35). Vancomycin, FC, rifampin, DAF-2DA, and C-PTIO potassium salt were purchased from Sigma-Aldrich (St. Louis, MO, USA).

### Bacterial strains.

P. aeruginosa PA14, P. aeruginosa PA14 (pUCP18), and P. aeruginosa PA14 mutants (Δ*wspF*, Δ*nasA*, Δ*nirS*, Δ*lasR*, and Δ*rhlR*) were provided by Y. H. Cho (Cha University, Seoul, Republic of Korea); E. coli K-12 BW25113 and the Keio E. coli knockout library were from the National Institute of Genetics (Shizuoka, Japan).

### Biofilm assay.

P. aeruginosa and E. coli biofilms were assayed in a 96-well polystyrene microtiter plate as previously described (17, 53).

### Quantitative analysis of EPS in biofilms.

EPS in biofilms of P. aeruginosa was evaluated as previously described (54).

### Confocal laser scanning microscopy for biofilm visualization and intracellular NO detection.

Confocal laser scanning microscopy for biofilm visualization (55) and intracellular NO detection (56) was performed as previously described with some modifications.

### Measurement of QS signaling molecules.

The effects of complestatin on the production of QS signaling molecules were determined as previously described (57).

### Quantitative cellular c-di-GMP analysis by liquid chromatography-tandem mass spectrometry.

c-di-GMP in P. aeruginosa and E. coli biofilms was analyzed using a previously described method (17).

### PDE and DGC activity assays.

The DGC and PDE activities in P. aeruginosa and E. coli were evaluated as described previously (17).

### Screening the E. coli Keio collection library.

Keio mutants representing 3,801 genes were screened to identify the specific target protein of complestatin.

### Overexpression assay.

Overexpression E. coli strains were constructed using the pBAD-TOPO TA expression vector.

### Expression and RT-qPCR of the nitrite reductase gene and related genes.

The transcription of the nitrite reductase gene and related genes was determined as previously described with some modifications (17).

### C. elegans virulence assay.

A C. elegans viability assay was executed as previously reported (17, 58).

### Statistical analysis.

Data are expressed as the means ± standard deviations (SDs). The unpaired Student's *t* test was used to analyze the data (Excel software; Microsoft, Redmond, WA, USA). A *P* value of <0.05 was considered statistically significant.

## References

[1] Flemming HC, Wingender J 2010 The biofilm matrix. Nat Rev Microbiol 8:623–633. doi:10.1038/nrmicro2415. 20676145

[2] O'Toole G, Kaplan HB, Kolter R 2000 Biofilm formation as microbial development. Annu Rev Microbiol 54:49–79. doi:10.1146/annurev.micro.54.1.49. 11018124

[3] Flemming HC, Neu TR, Wozniak DJ 2007 The EPS matrix: the “house of biofilm cells”. J Bacteriol 189:7945–7947. doi:10.1128/JB.00858-07. 17675377 PMC2168682

[4] Bjarnsholt T, Ciofu O, Molin S, Givskov M, Hoiby N 2013 Applying insights from biofilm biology to drug development - can a new approach be developed? Nat Rev Drug Discov 12:791–808. doi:10.1038/nrd4000. 24080700

[5] Davies D 2003 Understanding biofilm resistance to antibacterial agents. Nat Rev Drug Discov 2:114–122. doi:10.1038/nrd1008. 12563302

[6] World Health Organization. 2019 Antimicrobial resistance. World Health Organization, Geneva, Switzerland https://www.who.int/news-room/fact-sheets/detail/antimicrobial-resistance.

[7] Donlan RM, Costerton JW 2002 Biofilms: survival mechanisms of clinically relevant microorganisms. Clin Microbiol Rev 15:167–193. doi:10.1128/cmr.15.2.167-193.2002. 11932229 PMC118068

[8] Lewis K 2008 Multidrug tolerance of biofilms and persister cells. Curr Top Microbiol Immunol 322:107–131. doi:10.1007/978-3-540-75418-3_6. 18453274

[9] Smith K, Hunter IS 2008 Efficacy of common hospital biocides with biofilms of multi-drug resistant clinical isolates. J Med Microbiol 57:966–973. doi:10.1099/jmm.0.47668-0. 18628497

[10] Koo H, Allan RN, Howlin RP, Stoodley P, Hall-Stoodley L 2017 Targeting microbial biofilms: current and prospective therapeutic strategies. Nat Rev Microbiol 15:740–755. doi:10.1038/nrmicro.2017.99. 28944770 PMC5685531

[11] World Health Organization. 2017 Global priority list of antibiotic-resistant bacteria to guide research, discovery, and development of new antibiotics. World Health Organization, Geneva, Switzerland.

[12] Driscoll JA, Brody SL, Kollef MH 2007 The epidemiology, pathogenesis and treatment of *Pseudomonas aeruginosa* infections. Drugs 67:351–368. doi:10.2165/00003495-200767030-00003. 17335295

[13] Papenfort K, Bassler BL 2016 Quorum sensing signal-response systems in Gram-negative bacteria. Nat Rev Microbiol 14:576–588. doi:10.1038/nrmicro.2016.89. 27510864 PMC5056591

[14] Lee J, Zhang L 2015 The hierarchy quorum sensing network in *Pseudomonas aeruginosa*. Protein Cell 6:26–41. doi:10.1007/s13238-014-0100-x. 25249263 PMC4286720

[15] Sakuragi Y, Kolter R 2007 Quorum-sensing regulation of the biofilm matrix genes (*pel*) of *Pseudomonas aeruginosa*. J Bacteriol 189:5383–5386. doi:10.1128/JB.00137-07. 17496081 PMC1951888

[16] Hengge R 2009 Principles of c-di-GMP signalling in bacteria. Nat Rev Microbiol 7:263–273. doi:10.1038/nrmicro2109. 19287449

[17] Kim B, Park JS, Choi HY, Yoon SS, Kim WG 2018 Terrein is an inhibitor of quorum sensing and c-di-GMP in *Pseudomonas aeruginosa*: a connection between quorum sensing and c-di-GMP. Sci Rep 8:8617. doi:10.1038/s41598-018-26974-5. 29872101 PMC5988783

[18] Hengge R, Grundling A, Jenal U, Ryan R, Yildiz F 2016 Bacterial signal transduction by cyclic di-GMP and other nucleotide second messengers. J Bacteriol 198:15–26. doi:10.1128/JB.00331-15. 26055111 PMC4686208

[19] Romling U, Galperin MY, Gomelsky M 2013 Cyclic di-GMP: the first 25 years of a universal bacterial second messenger. Microbiol Mol Biol Rev 77:1–52. doi:10.1128/MMBR.00043-12. 23471616 PMC3591986

[20] Baraquet C, Harwood CS 2013 Cyclic diguanosine monophosphate represses bacterial flagella synthesis by interacting with the Walker A motif of the enhancer-binding protein FleQ. Proc Natl Acad Sci U S A 110:18478–18483. doi:10.1073/pnas.1318972110. 24167275 PMC3832005

[21] Ha DG, O’Toole GA 2015 c-di-GMP and its effects on biofilm formation and dispersion: a *Pseudomonas aeruginosa* review Microbiol Spectr 3:MB-0003-2014. doi:10.1128/microbiolspec.MB-0003-2014. PMC4498269 26104694

[22] McDougald D, Rice SA, Barraud N, Steinberg PD, Kjelleberg S 2011 Should we stay or should we go: mechanisms and ecological consequences for biofilm dispersal. Nat Rev Microbiol 10:39–50. doi:10.1038/nrmicro2695. 22120588

[23] Cutruzzola F, Frankenberg-Dinkel N 2016 Origin and impact of nitric oxide in *Pseudomonas aeruginosa* biofilms. J Bacteriol 198:55–65. doi:10.1128/JB.00371-15. 26260455 PMC4686190

[24] Arora DP, Hossain S, Xu Y, Boon EM 2015 Nitric oxide regulation of bacterial biofilms. Biochemistry 54:3717–3728. doi:10.1021/bi501476n. 25996573

[25] Zumft WG 1997 Cell biology and molecular basis of denitrification. Microbiol Mol Biol Rev 61:533–616. doi:10.1128/.61.4.533-616.1997. 9409151 PMC232623

[26] Hassett DJ, Cuppoletti J, Trapnell B, Lymar SV, Rowe JJ, Yoon SS, Hilliard GM, Parvatiyar K, Kamani MC, Wozniak DJ, Hwang SH, McDermott TR, Ochsner UA 2002 Anaerobic metabolism and quorum sensing by *Pseudomonas aeruginosa* biofilms in chronically infected cystic fibrosis airways: rethinking antibiotic treatment strategies and drug targets. Adv Drug Deliv Rev 54:1425–1443. doi:10.1016/s0169-409x(02)00152-7. 12458153

[27] Ojoo JC, Mulrennan SA, Kastelik JA, Morice AH, Redington AE 2005 Exhaled breath condensate pH and exhaled nitric oxide in allergic asthma and in cystic fibrosis. Thorax 60:22–26. doi:10.1136/thx.2003.017327. 15618578 PMC1747154

[28] Zhou G, Peng H, Wang YS, Li CL, Shen PF, Huang XM, Xie XB, Shi QS 2019 Biological functions of *nirS* in *Pseudomonas aeruginosa* ATCC 9027 under aerobic conditions. J Ind Microbiol Biotechnol 46:1757–1768. doi:10.1007/s10295-019-02232-z. 31512096

[29] Rabin N, Zheng Y, Opoku-Temeng C, Du Y, Bonsu E, Sintim HO 2015 Biofilm formation mechanisms and targets for developing antibiofilm agents. Future Med Chem 7:493–512. doi:10.4155/fmc.15.6. 25875875

[30] Barraud N, Hassett DJ, Hwang SH, Rice SA, Kjelleberg S, Webb JS 2006 Involvement of nitric oxide in biofilm dispersal of *Pseudomonas aeruginosa*. J Bacteriol 188:7344–7353. doi:10.1128/JB.00779-06. 17050922 PMC1636254

[31] Barraud N, Schleheck D, Klebensberger J, Webb JS, Hassett DJ, Rice SA, Kjelleberg S 2009 Nitric oxide signaling in *Pseudomonas aeruginosa* biofilms mediates phosphodiesterase activity, decreased cyclic di-GMP levels, and enhanced dispersal. J Bacteriol 191:7333–7342. doi:10.1128/JB.00975-09. 19801410 PMC2786556

[32] Yoon MY, Lee KM, Park Y, Yoon SS 2011 Contribution of cell elongation to the biofilm formation of *Pseudomonas aeruginosa* during anaerobic respiration. PLoS One 6:e16105. doi:10.1371/journal.pone.0016105. 21267455 PMC3022656

[33] Barraud N, Kelso MJ, Rice SA, Kjelleberg S 2015 Nitric oxide: a key mediator of biofilm dispersal with applications in infectious diseases. Curr Pharm Des 21:31–42. doi:10.2174/1381612820666140905112822. 25189865

[34] Kaneko I, Kamoshida K, Takahashi S 1989 Complestatin, a potent anti-complement substance produced by *Streptomyces lavendulae*. I. Fermentation, isolation and biological characterization. J Antibiot (Tokyo) 42:236–241. doi:10.7164/antibiotics.42.236. 2925515

[35] Kwon YJ, Kim HJ, Kim WG 2015 Complestatin exerts antibacterial activity by the inhibition of fatty acid synthesis. Biol Pharm Bull 38:715–721. doi:10.1248/bpb.b14-00824. 25947917

[36] Chung IY, Choi KB, Heo YJ, Cho YH 2008 Effect of PEL exopolysaccharide on the *wspF* mutant phenotypes in *Pseudomonas aeruginosa* PA14. J Microbiol Biotechnol 18:1227–1234. 18667850

[37] Kalia D, Merey G, Nakayama S, Zheng Y, Zhou J, Luo Y, Guo M, Roembke BT, Sintim HO 2013 Nucleotide, c-di-GMP, c-di-AMP, cGMP, cAMP, (p)ppGpp signaling in bacteria and implications in pathogenesis. Chem Soc Rev 42:305–341. doi:10.1039/c2cs35206k. 23023210

[38] Baba T, Ara T, Hasegawa M, Takai Y, Okumura Y, Baba M, Datsenko KA, Tomita M, Wanner BL, Mori H 2006 Construction of *Escherichia coli* K-12 in-frame, single-gene knockout mutants: the Keio collection. Mol Syst Biol 2:2006.0008. doi:10.1038/msb4100050. PMC1681482 16738554

[39] Corker H, Poole RK 2003 Nitric oxide formation by *Escherichia coli*. Dependence on nitrite reductase, the NO-sensing regulator Fnr, and flavohemoglobin Hmp. J Biol Chem 278:31584–31592. doi:10.1074/jbc.M303282200. 12783887

[40] Weiss B 2006 Evidence for mutagenesis by nitric oxide during nitrate metabolism in *Escherichia coli*. J Bacteriol 188:829–833. doi:10.1128/JB.188.3.829-833.2006. 16428385 PMC1347335

[41] Derbyshire ER, Marletta MA 2012 Structure and regulation of soluble guanylate cyclase. Annu Rev Biochem 81:533–559. doi:10.1146/annurev-biochem-050410-100030. 22404633

[42] Kang D, Kirienko NV 2017 High-throughput genetic screen reveals that early attachment and biofilm formation are necessary for full pyoverdine production by *Pseudomonas aeruginosa*. Front Microbiol 8:1707. doi:10.3389/fmicb.2017.01707. 28928729 PMC5591869

[43] Darby C, Cosma CL, Thomas JH, Manoil C 1999 Lethal paralysis of *Caenorhabditis elegans* by *Pseudomonas aeruginosa*. Proc Natl Acad Sci U S A 96:15202–15207. doi:10.1073/pnas.96.26.15202. 10611362 PMC24797

[44] Tan MW, Ausubel FM 2000 *Caenorhabditis elegans*: a model genetic host to study *Pseudomonas aeruginosa* pathogenesis. Curr Opin Microbiol 3:29–34. doi:10.1016/S1369-5274(99)00047-8. 10679415

[45] Tan MW, Mahajan-Miklos S, Ausubel FM 1999 Killing of *Caenorhabditis elegans* by *Pseudomonas aeruginosa* used to model mammalian bacterial pathogenesis. Proc Natl Acad Sci U S A 96:715–720. doi:10.1073/pnas.96.2.715. 9892699 PMC15202

[46] Utari PD, Quax WJ 2013 *Caenorhabditis elegans* reveals novel *Pseudomonas aeruginosa* virulence mechanism. Trends Microbiol 21:315–316. doi:10.1016/j.tim.2013.04.006. 23684150

[47] Martinez-Espinosa RM, Cole JA, Richardson DJ, Watmough NJ 2011 Enzymology and ecology of the nitrogen cycle. Biochem Soc Trans 39:175–178. doi:10.1042/BST0390175. 21265768

[48] Einsle O, Kroneck PM 2004 Structural basis of denitrification. Biol Chem 385:875–883. doi:10.1515/BC.2004.115. 15551861

[49] Worlitzsch D, Tarran R, Ulrich M, Schwab U, Cekici A, Meyer KC, Birrer P, Bellon G, Berger J, Weiss T, Botzenhart K, Yankaskas JR, Randell S, Boucher RC, Doring G 2002 Effects of reduced mucus oxygen concentration in airway *Pseudomonas* infections of cystic fibrosis patients. J Clin Invest 109:317–325. doi:10.1172/JCI13870. 11827991 PMC150856

[50] Arai H 2011 Regulation and function of versatile aerobic and anaerobic respiratory metabolism in *Pseudomonas aeruginosa*. Front Microbiol 2:103. doi:10.3389/fmicb.2011.00103. 21833336 PMC3153056

[51] Schreiber K, Krieger R, Benkert B, Eschbach M, Arai H, Schobert M, Jahn D 2007 The anaerobic regulatory network required for *Pseudomonas aeruginosa* nitrate respiration. J Bacteriol 189:4310–4314. doi:10.1128/JB.00240-07. 17400734 PMC1913380

[52] Arai H, Kodama T, Igarashi Y 1997 Cascade regulation of the two CRP/FNR-related transcriptional regulators (ANR and DNR) and the denitrification enzymes in *Pseudomonas aeruginosa*. Mol Microbiol 25:1141–1148. doi:10.1046/j.1365-2958.1997.5431906.x. 9350869

[53] O’Toole GA 2011 Microtiter dish biofilm formation assay. J Vis Exp 2011:2437. doi:10.3791/2437. PMC3182663 21307833

[54] Thomann A, de Mello Martins AG, Brengel C, Empting M, Hartmann RW 2016 Application of dual inhibition concept within looped autoregulatory systems toward antivirulence agents against *Pseudomonas aeruginosa* infections. ACS Chem Biol 11:1279–1286. doi:10.1021/acschembio.6b00117. 26882081

[55] Li H, Li X, Wang Z, Fu Y, Ai Q, Dong Y, Yu J 2015 Autoinducer-2 regulates *Pseudomonas aeruginosa* PAO1 biofilm formation and virulence production in a dose-dependent manner. BMC Microbiol 15:192. doi:10.1186/s12866-015-0529-y. 26420312 PMC4588260

[56] Slomberg DL, Lu Y, Broadnax AD, Hunter RA, Carpenter AW, Schoenfisch MH 2013 Role of size and shape on biofilm eradication for nitric oxide-releasing silica nanoparticles. ACS Appl Mater Interfaces 5:9322–9329. doi:10.1021/am402618w. 24006838

[57] Kim B, ParK J-S, Choi H-Y, Kwak J-H, Kim W-G 2019 Differential effects of alkyl gallates on quorum sensing in *Pseudomonas aeruginosa*. Sci Rep 9:7741. doi:10.1038/s41598-019-44236-w. 31123307 PMC6533263

[58] O'Loughlin CT, Miller LC, Siryaporn A, Drescher K, Semmelhack MF, Bassler BL 2013 A quorum-sensing inhibitor blocks *Pseudomonas aeruginosa* virulence and biofilm formation. Proc Natl Acad Sci U S A 110:17981–17986. doi:10.1073/pnas.1316981110. 24143808 PMC3816427

