# Supplementary figures and images for: The Nitrite Transporter Facilitates Biofilm Formation via Suppression of Nitrite Reductase and Is a New Antibiofilm Target in Pseudomonas aeruginosa
Source: mBio. 2020 Jul 7;11(4):e00878-20. doi: 10.1128/mBio.00878-20 (PMC7343986; doi:10.1128/mBio.00878-20)

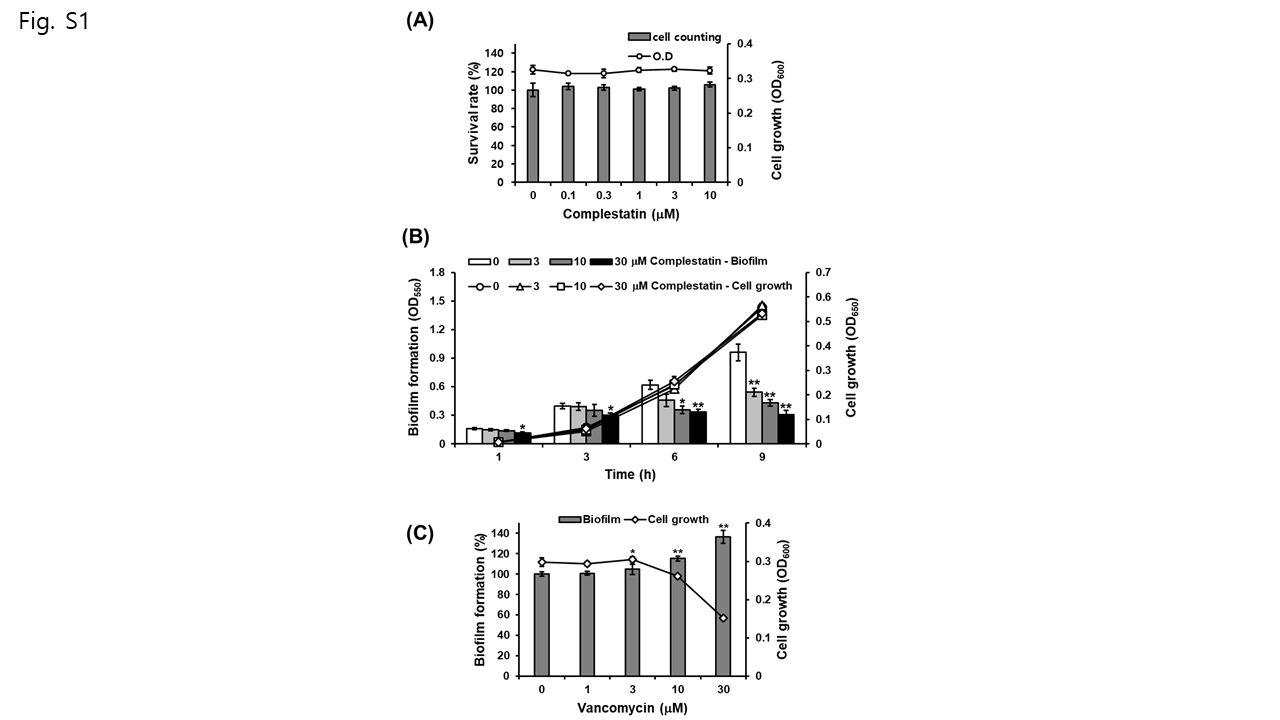

Supplement: FIG S1 [file mBio.00878-20-sf001.tif]

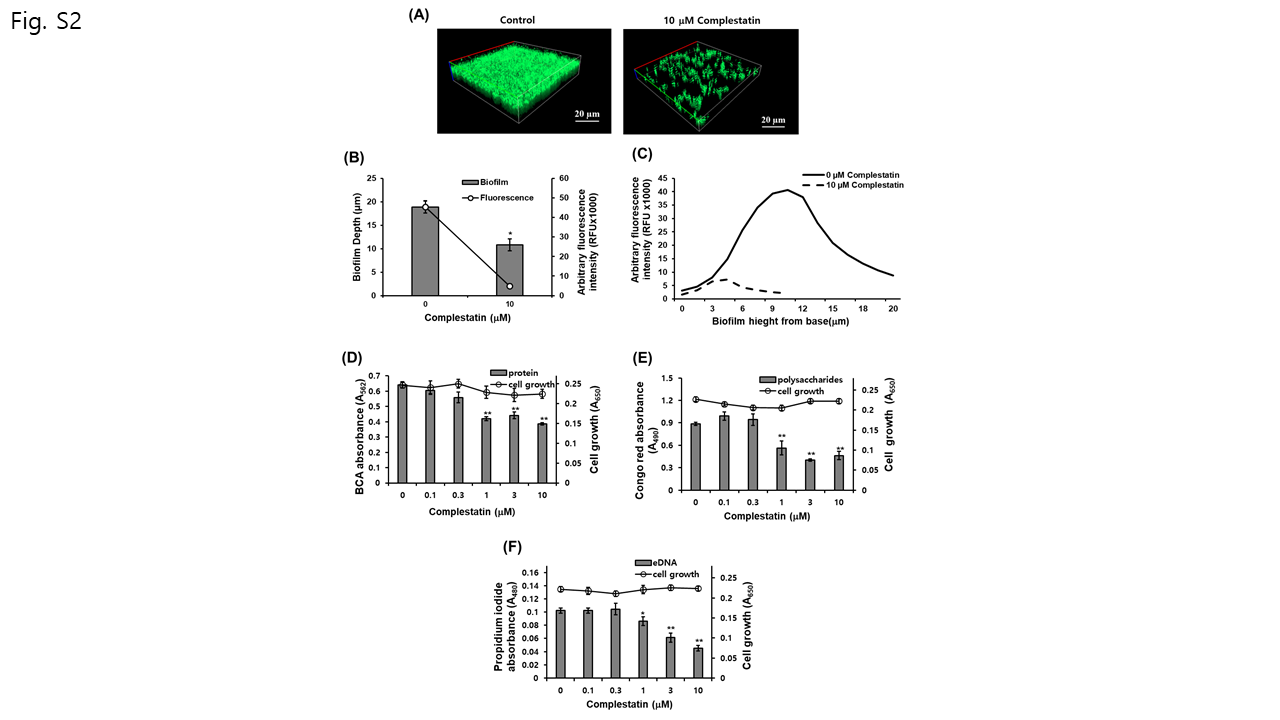

Supplement: FIG S2 [file mBio.00878-20-sf002.tif]

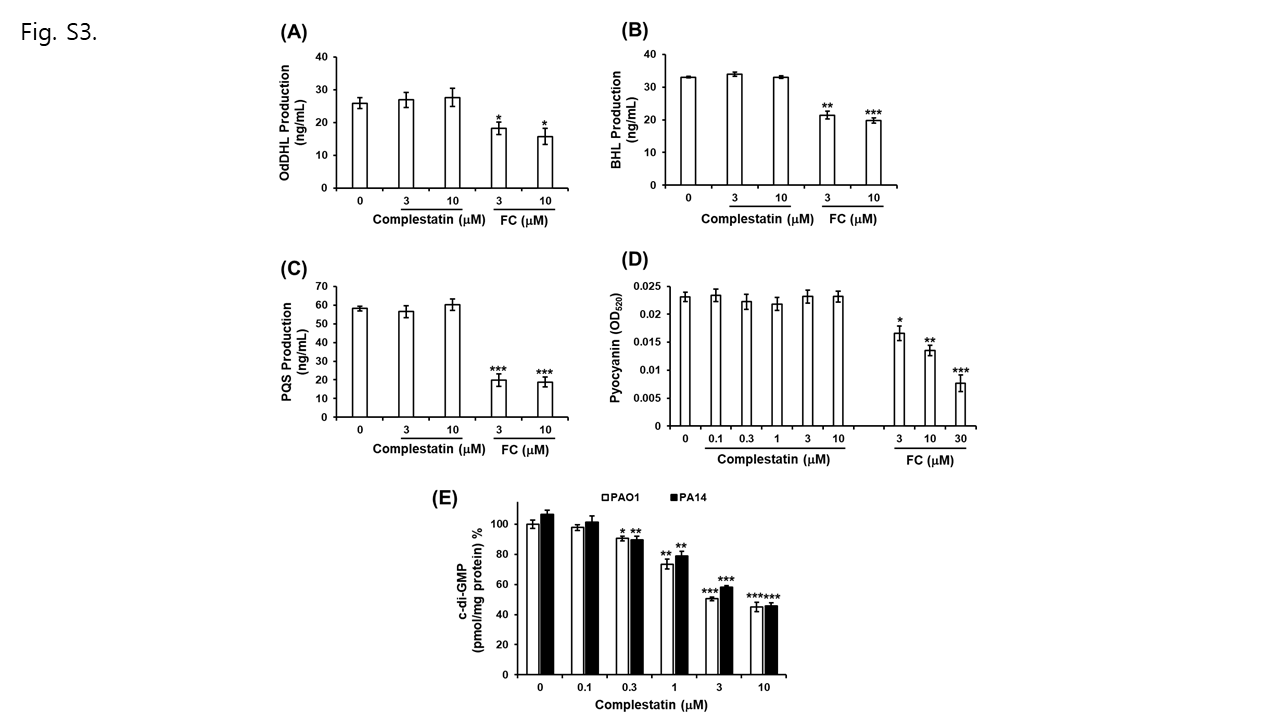

Supplement: FIG S3 [file mBio.00878-20-sf003.tif]

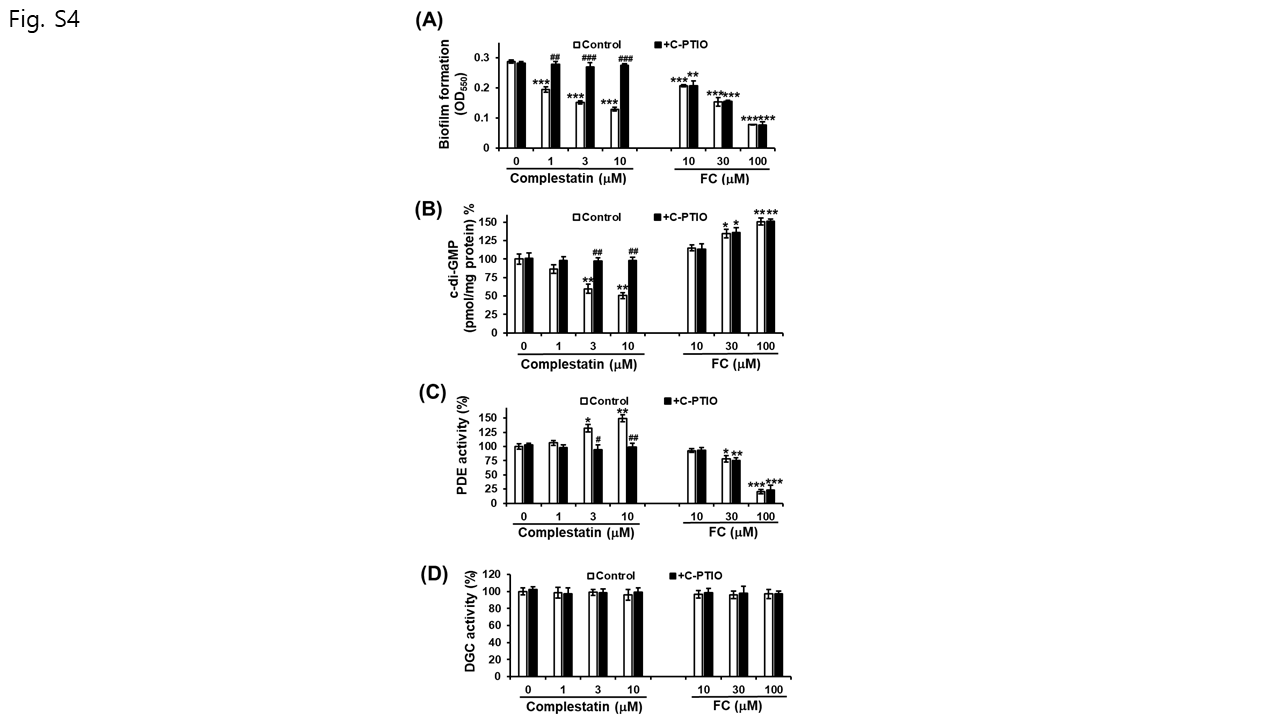

Supplement: FIG S4 [file mBio.00878-20-sf004.tif]

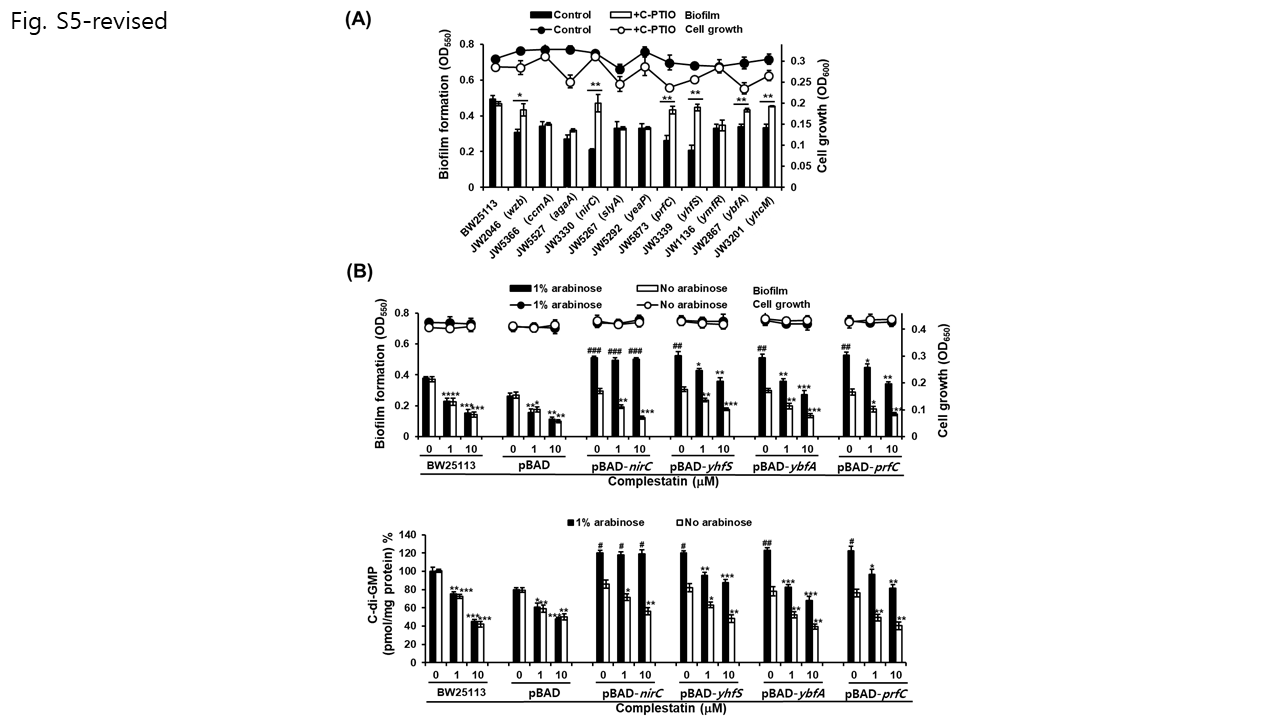

Supplement: FIG S5 [file mBio.00878-20-sf005.tif]

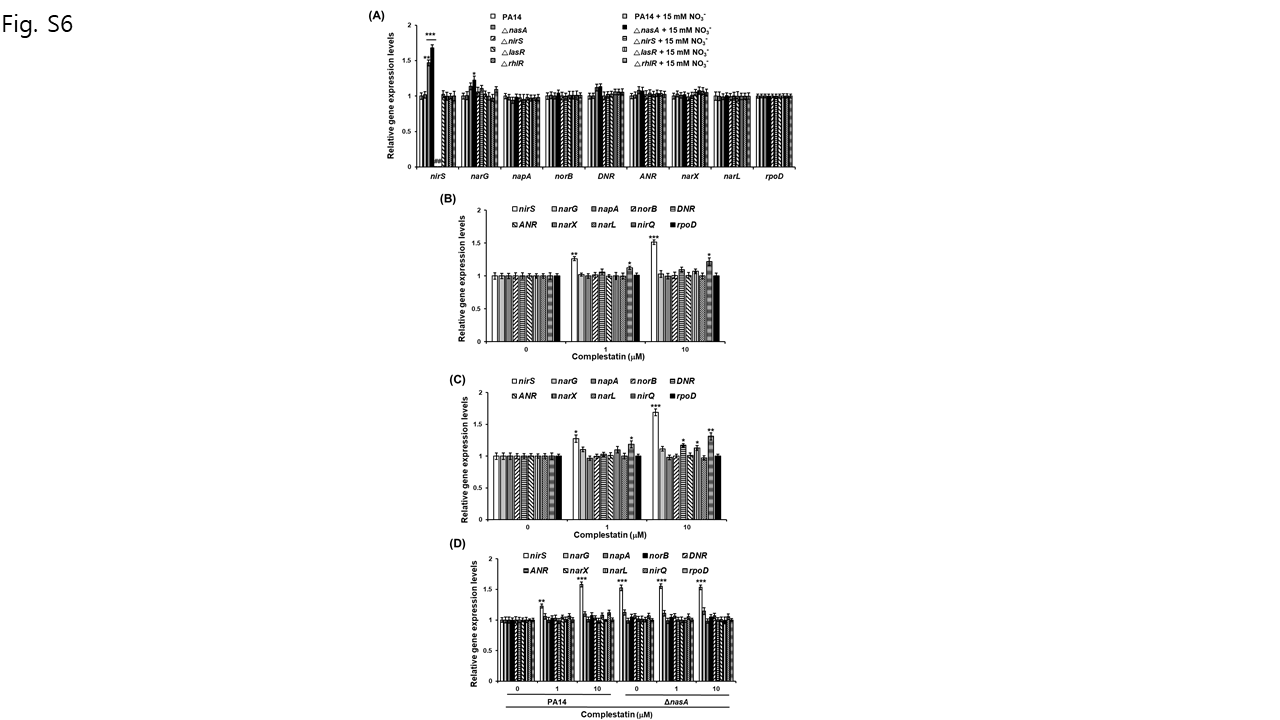

Supplement: FIG S6 [file mBio.00878-20-sf006.tif]

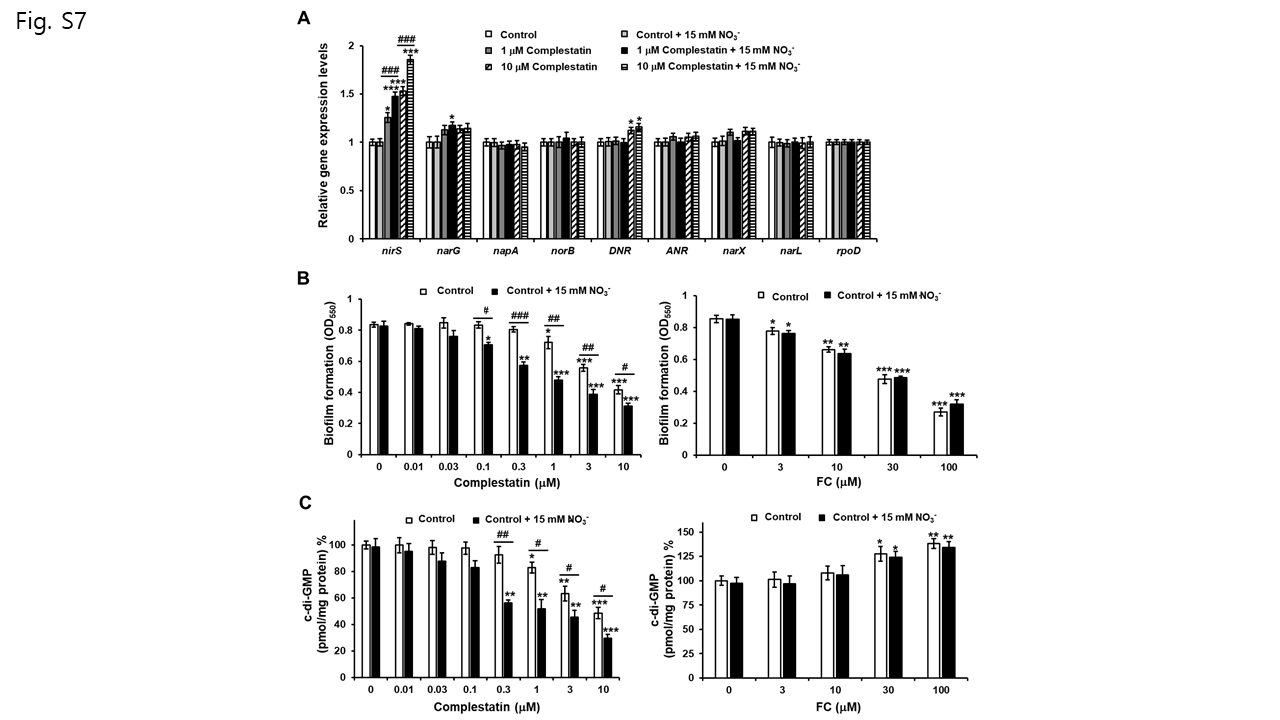

Supplement: FIG S7 [file mBio.00878-20-sf007.tif]

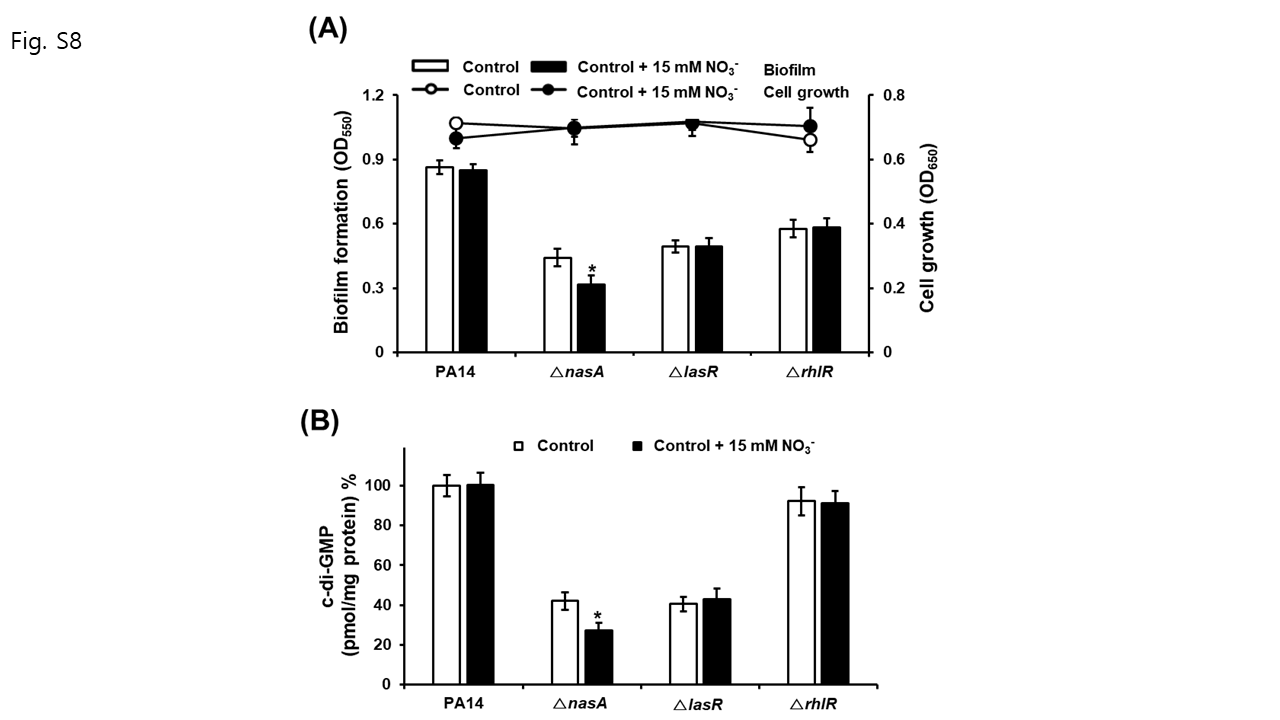

Supplement: FIG S8 [file mBio.00878-20-sf008.tif]
