# Supplementary material for: The Nitrite Transporter Facilitates Biofilm Formation via Suppression of Nitrite Reductase and Is a New Antibiofilm Target in Pseudomonas aeruginosa
Source: mBio. 2020 Jul 7;11(4):e00878-20. doi: 10.1128/mBio.00878-20 (PMC7343986; doi:10.1128/mBio.00878-20)
Supplement: TABLE S1 [file mBio.00878-20-st001.pdf]

Table S1.

Gene and primer type	Gene product and PCR primer sequence (5' to 3')
prfC	Peptide chain release factor
Forward	ATGACGTTGTCTCCTTATTTGCAAG
Reverse	TTAATGCTCGCGGGTCTGGTGGAAC
yhfS	Uncharacterized protein
Forward	ATGAAGACGTTTCCTCTGCAAAGCC
Reverse	TTAAATACTGGCAATACTCTCACGC
nirC	Nitrite transporter
Forward	ATGTTTACAGACACTATTAATAAGT
Reverse	TTAACCGGCAGCCGTTTCAGTTTGA
ybfA	Uncharacterized protein
Forward	ATGGAACTCTACAGAGAATATCCTG
Reverse	TCAATAAAAATCACCAGTTGCCTTT
