# Supplementary material for: The Nitrite Transporter Facilitates Biofilm Formation via Suppression of Nitrite Reductase and Is a New Antibiofilm Target in Pseudomonas aeruginosa
Source: mBio. 2020 Jul 7;11(4):e00878-20. doi: 10.1128/mBio.00878-20 (PMC7343986; doi:10.1128/mBio.00878-20)
Supplement: TEXT S1 [file mBio.00878-20-s0001.docx]

**Supplemental Materials and Methods**

**Biofilm assay.** *P. aeruginosa* and *E. coli* biofilms were assayed in a 96-well polystyrene microtiter plate as previously described (1, 2). *P. aeruginosa* or *E. coli* cells were cultured overnight and diluted 100-fold using M63 medium. Diluted cell suspensions (100 μL) were dispensed in 20 or more wells of a 96-well polystyrene microplate. DMSO as a solvent control or test compounds were added to the wells. *P. aeruginosa* and *E. coli* cells in the wells were statically incubated for 9 h and 18 h, respectively, at 37 °C. After the planktonic cell viability was measured by either measuring the optical density (OD) at 600 nm or counting viable cells, unattached cells and media were discarded, and the biofilms, which were attached to the surface of the wells, were stained using 120 μL of 0.1% crystal violet for 10 min. Stained cells were washed with deionized water to remove unbound crystal violet. The bound crystal violet was solubilized with 150 μL of 30% aqueous acetic acid for 15 min. The OD of the eluted crystal violet was measured at 550 nm by a microplate reader. The inhibitory effect of vancomycin, on biofilm formation and planktonic cell growth was also determined by the same procedure. For a pre-established biofilm assay, biofilms were pre-formed by incubating for 9 h and, then after changing to a fresh media, treated with test compounds for 12 h. A PA14 (pUCP18) vector control and *P*. *aeruginosa wspF* mutant were incubated for 9 h in M63 medium containing 50 μg/mL rifampicin in the presence of FC or different concentrations of complestatin with the same procedure to compare the ability of both strains to form biofilms in the presence of complestatin.

**Viable cell counting.** *P. aeruginosa* PA14 overnight cultures were diluted 100–times in M63 medium in falcon tube. Cell suspensions (100 μL) were dispensed in wells of a 96-well polystyrene microplate. Biofilms were grown in static condition for 9 h incubation in a 96-well polystyrene microplate in presence of various concentration of complestatin. The unattached cells were collected by centrifugation at 12,000 rpm at 4°C for 10 min to obtain planktonic cells. Bacterial cells inside the biofilms were disengaged by sonicating for 2 min at a frequency of 40 KHz with a 50 W power output. Viable cells of both types (planktonic and biofilm) were counted by serial-dilution and plating.

**Quantitative analysis of extracellular polymeric substances (EPS) in biofilms.** EPS in biofilms of *P. aeruginosa* was evaluated as previously described (3). *P. aeruginosa* PA14 were incubated in 96-well plate for 9 h at 37°C in M63 medium with complestatin-supplementation. After the measurement of planktonic cells in each well at 600 nm, the unattached cells and medium was discarded and the inner-surface of each well was washed with PBS to remove loosely associated bacteria. For analysis of polysaccharides, the biofilms were stained with 0.2 mL of Congo red solution (20 mg/mL) for 3h at 37°C. Stained cells were washed with deionized water and was solubilized with 0.2 mL of 95% ethanol for 10 min. The optical density of the eluted crystal violet was measured at 490 nm. For the detection of protein level, after the biofilms were treated with 0.2 mL of BCA protein assay reagent and incubated for 3h at 37°C, the absorbance at 562 nm was measured. eDNA levels in the biofilms was assessed by staining with 0.1 mL of propidium iodide (0.05 mg/mL) for 3h at 37°C and measuring the absorbance at 480 nm after washing twice with deionized water.

**Confocal laser scanning microscopy for biofilm visualization and intracellular NO detection.** Confocal laser scanning microscopy for biofilm visualization (4) and intracellular NO detection (5) was performed as previously described with some modifications. *P. aeruginosa* or *E. coli* biofilms were grown on 15-mm^2^ glass coverslips (Matsunami Glass Ind., Ltd. Japan). Sterile coverslips were positioned vertically in 24-well plates. *P. aeruginosa* and *E. coli* were statically incubated at 37 °C for 6 h and 18 h, respectively. Coverslips were washed twice with sterile phosphate-buffered saline (PBS), and the biofilms on the coverslips were then stained with SYTO9/propidium iodide following the LIVE/DEAD BacLight Bacterial Viability Kit (Invitrogen Molecular Probes, USA) instructions. After staining, biofilms were washed with sterile PBS to remove planktonic bacteria and dyes. The biofilms were then visualized by CLSM (Carl Zeiss LSM800, Jena, Germany) by excitation at 543 nm (emission: 600 nm) and detection at 488 nm (emission: 515 nm). For the intracellular NO detection, the biofilms on the coverslips were stained with the fluorescent NO probe DAF-2DA (20 μM, Sigma) for 1 h. After washing biofilms with sterile PBS, the biofilms were then excited at 480 nm (emission: 515 nm).

**Measurement of QS signaling molecules.** The effects of complestatin on the production of QS signaling molecules were determined as previously described (6). Overnight cultures of *P. aeruginosa* PA14 were diluted 100–fold in LB medium, and 1 mL of culture was dispensed into 15-mL conical tubes, treated with test compound dissolved in DMSO, and incubated at 220 rpm at 37°C for 24 h. The cultures were then centrifuged at 12,000 rpm at 4°C for 10 min. The three QS signaling molecules, OdDHL, BHL, and PQS, were extracted from 2 mL of culture supernatants. Extraction was performed with 2 mL of ethyl acetate, which was acidified with 0.1% acetic acid, and the organic layer was evaporated under a vacuum and quantified by LC-MS/MS in MRM mode. The samples were subjected to an HPLC system (Luna C18(2), 100 × 2.0 mm, 3 μm, Phenomenex, Torrance, CA, USA) connected to a QTrap 3200 with a Turbolon Spray source (AB SCIEX, Singapore).

**Pyocyanin assay.** The production of pyocyanin in *P. aeruginosa* was assayed as previously described (2). For pyocyanin production, after overnight cultures of *P. aeruginosa* PA14 were diluted 100–fold in LB medium, 5 mL of culture was dispensed into 50 mL conical tubes, treated with test compounds dissolved in DMSO, and incubated at 220 rpm at 37°C for 24 h. The cultures were then centrifuged at 12,000 rpm at 4°C for 10 min. 5 mL of supernatant was mixed with 3 mL of chloroform and 2 mL of 0.2 N HCl was added to the chloroform fraction. The resultant aqueous fraction was measured at 520 nm by a microplate reader.

**Quantitative cellular c-di-GMP analysis by LC-MS/MS.** C-di-GMP in *P. aeruginosa* and *E. coli* biofilms was analyzed using a previously described method (2). *P. aeruginosa* and *E. coli* were cultured overnight, and the cultures were diluted 100-fold in M63 medium to determine the c-di-GMP concentrations in biofilm cells. The diluted cultures (100 μL) were then dispensed in wells of 96-well plates. Different concentrations of complestatin or DMSO as a solvent control were supplemented to treat the cells. *P. aeruginosa* and *E. coli* cells were statically incubated at 37 °C for 9 h and 18 h, respectively, in the presence of drugs. Unattached cells and medium were discarded, and surface-attached biofilm cells were rinsed with distilled water. After washing the biofilm cells, 100 μL of M63 medium was dispensed in each well. The 96-well plates were sonicated for 2 min to detach the biofilm cells from the wells, and the cells were collected in a tube. The tubes were centrifuged at 4 °C for 10 min at 12,000 × g, and the cells were harvested. The cells were then suspended again in M63 medium, and 70% v/v perchloric acid was added to the biofilm cell suspension, where the final perchloric acid concentration in the cell suspension was 0.6 M. Initially, the perchloric acid-supplemented biofilm cell suspensions were incubated for 30 min on ice, and then the rest of the experimental phases were performed at 4 °C. These cell suspensions were centrifuged for 10 min at 12000 × g, maintaining a temperature of 4 °C. The cell suspension supernatants were collected in 1.5-mL Eppendorf tubes. A Pierce BCA protein assay kit (Thermo Scientific, Waltham, MA, USA) was used to measure the protein concentration in the precipitates. To neutralize the pH of the nucleotide extracts, 20 μL of potassium bicarbonate solution (2.5 M) was added. Then, the mixtures were briefly centrifuged to collect the samples. The nucleotide extract supernatants were then transferred to fresh 1.5-mL Eppendorf tubes, And the tubes were centrifuged for 10 min to remove perchlorate salt precipitates. LC-MS/MS was utilized to measure the c-di-GMP concentrations in the supernatants. The samples were subjected to an HPLC system (Luna C18(2), 100 × 2.0 mm, 3 μm, Phenomenex, Torrance, CA, USA) connected to a QTrap 3200 with a Turbolon Spray source (AB SCIEX, Singapore).

**PDE and DGC activity assays.** The DGC and PDE activities in *P*. *aeruginosa* and *E. coli* were evaluated as described previously (2). *P*. *aeruginosa* and *E. coli* overnight cultures were diluted 100-fold using LB medium as the diluent. Different concentrations of test compounds were added to the diluted cultures and incubated for 6 h and 24 h in *P. aeruginosa* and *E. coli* cultures, respectively, at 37 °C and 220 rpm in a shaking incubator. The cultures were centrifuged for 10 min at 12000 × g, maintaining a temperature of 4 °C. The harvested cells were resuspended by adding 1 mL of LB medium. Two different types of buffers (200 μL) were added separately to the harvested cell suspensions for evaluating DGC and PDE activities. The buffer composition for the PDE activity assay was 50 mM Tris base (pH 8.1), 50 mM sodium chloride, 1 mM manganese chloride, and 5 mM bis-para-nitrophenyl phosphate (pNPP). Bis-pNPP is generally known as an artificial PDE substrate that is particularly associated with c-di-GMP. The buffer used for the DGC activity assay was composed of 250 mM sodium chloride, 75 mM Tris-hydrochloride (pH 7.8), 25 mM potassium chloride and 10 mM magnesium sulfate. The cells were suspended in buffer, lysed by sonicating 10 times, for 30 sec each time. The lysed cell suspensions were kept in a shaking incubator for 2 h at 37 °C with a rotation frequency of 60 rpm for the PDE reaction. p-Nitrophenol formation from bis-pNPP was then determined by an ELISA reader at a wavelength of 410 nm. Twenty-five micromoles of GTP, as the substrate, was added to the supernatants to determine DGC activity. The mixture was then incubated for 2 h at 37 °C at 60 rpm in a shaking incubator. Cell lysates with no GTP accumulation were considered blanks. LC-MS/MS was used to quantify the concentration of c-di-GMP, which was synthesized by the interaction of DGC enzymes with GTP. Pierce BCA protein assays were performed to measure the total protein concentration, which was used to normalize the PDE (OD at 410 nm) and DGC (c-di-GMP in pmol) activities.

**Screening the *E. coli* Keio collection library.** Keio mutants representing 3801 genes were cultured overnight in 96-well plates containing 100 μL of LB medium supplemented with 15 μg/mL kanamycin. Then, 3 μL of the overnight culture was added to 100 μL of M63 medium in 96-well plates and grown for 18 h at 37 °C. After the planktonic cell viability was determined by either measuring the OD at 600 nm or by counting viable cells, biofilm assays were performed. The primary hits showing at least 30% less biofilm than the Keio parent strain *E. coli* BW25113 were rescreened three times. The biofilm-forming ability of the primary hits was reassayed in the presence of C-PTIO, where hits showing enhanced biofilm production were selected as secondary hits. The intracellular c-di-GMP levels and PDE activity of the secondary hits were assayed, and hits showing lower c-di-GMP levels and more PDE activity than BW25113 were selected as the final hits for an overexpression assay.

**Overexpression assay.** Four overexpression *E. coli* strains were constructed. The wild-type *nirC, yhfS*, *ybfA*, and *prfC* genes from *E. coli* W3110 genomic DNA were amplified by PCR using the primers listed in Table S1A. Each resulting products were cloned into the pBAD-TOPO TA expression vector (Invitrogen, Carlsbad, CA, USA), yielding respective pBAD recombinant plasmids that expressed under the control of the arabinose promoter (7). Each recombinant pBAD plasmids were then introduced into *E. coli* BW25113 via electroporation to produce respective overexpression strains. For the *nirC* gene, recombinant pBAD-*nirC* was introduced into *E. coli* BW25113 and *E. coli nirC* mutant to produce *E. coli* BW25113 (pBAD-*nirC*) and *E. coli* Δ*nirC* (pBAD-*nirC*), respectively.

**Expression and RT-qPCR of the nitrite reductase gene and related genes.** The transcription of the nitrite reductase gene and related genes were determined as previously described with some modifications (2). Overnight *P. aeruginosa* and *E. coli* cultures were diluted 100–fold in LB medium, and 1 mL of culture was dispensed into 15-mL conical tubes, treated with test compounds dissolved in DMSO, and incubated at 220 rpm at 37 °C for 12 h. The cultures were then centrifuged at 12,000 rpm at 4 °C for 10 min. Total RNA was extracted using TRIzol reagent (Invitrogen) according to the manufacturer’s instructions. cDNA for RT-qPCR detection of the expression of target genes was synthesized, and RT-qPCR using a Bio-Rad CFX-96 real time system (Bio-Rad, Hercules, CA, USA) was then performed. mRNA expression was normalized using the endogenous *rpoD* gene*.*

Genes and Primers used for quantitative RT-PCR in *E. coli* are as follows; nirB (forward: 5’-CGTTTACCTCGACGAAAGTA-3’ and reverse: 5’- TTTTCCGGCAGTTCGATAGC-3’), nrfA (forward: 5’- ACGGCAAAAACAAAGCGGTT -3’ and reverse: 5’- TTTCATATTCCGGGTGCTGC -3’), narG (forward: 5’- AAACCCAGCAGACTGACTAT -3’ and reverse: 5’- CATTTTCATCAGGCGTTTGC -3’), napA (forward: 5’- AAGCCATCAAATGGGATAAA -3’ and reverse: 5’- GGAAATAGCCCTTAATGCAG -3’), norV (forward: 5’- ATCGTGATTAACCATGCAGA -3’ and reverse: 5’- ACCACATTAAAATTCCACTC -3’), FNR (forward: 5’- TTTGACGCCATCGGCAGC -3’ and reverse: 5’- TTCACCGCTCATCAGACGCA -3’), rpoD (forward: 5’- AGGAAGATCTGGACGATGAC -3’ and reverse: 5’- CTTTGATGGTGTCACGCGTT -3’).

Genes and Primers used for quantitative RT-PCR in *P. aeruginosa* are as follows; nirS (forward: 5’- AAAGCTCCAAGTTCAAGGGCTA -3’ and reverse: 5’- GGTGGTAGGTCTGGGTGTCTAC -3’), narG (forward: 5’- GACGAGGTCACCGAGATCAT -3’ and reverse: 5’- AGTCGTAGAAGCTCAGGCAGAC -3’), napA (forward: 5’- ACGTCACCAGCCTGGTCT -3’ and reverse: 5’- AGCCCTTCACGCAGTTGAT -3’), norB (forward: 5’- ATCAGCATGGTCCTGATGACC -3’ and reverse: 5’- CCCATGATCAGTTCCCACAC -3’), DNR (forward: 5’- CAGCTGTTCCGTTTCTCCAA -3’ and reverse: 5’- GCGGTGGGTGGCGTTCTT -3’), ANR (forward: 5’- GAATTCCTGTTCCGCCAGG -3’ and reverse: 5’- ATCCATCCCGGACAGGCC -3’), narX (forward: 5’- TAGTCCGCTTCGGCTGCTA -3’ and reverse: 5’- GGGCGACCCGGTAGGTGAG -3’), narL (forward: 5’- TTCGCCTGCTGCTGGTGGA -3’ and reverse: 5’- GGATCATGTCCGGGTCCAG -3’), nirQ (forward: 5’- CTGCCGGTATTGCTCAAGGG -3’ and reverse: 5’- GGCGCCGATCAGATGGCG -3’), rpoD (forward: 5’- GGGGATCAACGTATTCGAGA -3’ and reverse: 5’- GGTACCCATTTCACGCATGT -3’).

**C. *elegans* virulence assay.** A *C*. *elegans* viability assay was executed as previously reported (2, 8). In the presence of an *E*. *coli* OP50 lawn, *C*. *elegans* was spread on NGM plates for 48 h at 20 °C to reach the L4 stage. Five microliters of *P. aeruginosa* overnight cultures were spread on a Petri plate (35 mm) that contained 4 mL of PGS agar and was used as a killing plate. The control plate was made in the same manner as the killing plate, using *E*. *coli* OP50 cells rather than *P. aeruginosa* cells. The organisms on the plates were first incubated for 24 h at 37 °C and then incubated for 24 h at 23 °C. Thirty L4-stage worms were introduced on the plates and left at room temperature for 30 h to incubate. Every 5 h, the worms were checked for survival, and the survival rates were recorded. DMSO as a solvent control or the test compounds were added to the killing plates.**References:**

1. O'Toole GA. 2011. Microtiter dish biofilm formation assay. J Vis Exp doi:10.3791/2437.

2. Kim B, Park JS, Choi HY, Yoon SS, Kim WG. 2018. Terrein is an inhibitor of quorum sensing and c-di-GMP in Pseudomonas aeruginosa: a connection between quorum sensing and c-di-GMP. Sci Rep 8:8617.

3. Thomann A, de Mello Martins AG, Brengel C, Empting M, Hartmann RW. 2016. Application of Dual Inhibition Concept within Looped Autoregulatory Systems toward Antivirulence Agents against Pseudomonas aeruginosa Infections. ACS Chem Biol 11:1279-86.

4. Li H, Li X, Wang Z, Fu Y, Ai Q, Dong Y, Yu J. 2015. Autoinducer-2 regulates Pseudomonas aeruginosa PAO1 biofilm formation and virulence production in a dose-dependent manner. BMC Microbiol 15:192.

5. Slomberg DL, Lu Y, Broadnax AD, Hunter RA, Carpenter AW, Schoenfisch MH. 2013. Role of size and shape on biofilm eradication for nitric oxide-releasing silica nanoparticles. ACS Appl Mater Interfaces 5:9322-9.

6. Kim B, ParK J-S, Choi H-Y, Kwak J-H, Kim W-G. 2019. Differential effects of alkyl gallates on quorum sensing in Pseudomonas aeruginosa. Scientific Reports 9:7741.

7. Guzman LM, Belin D, Carson MJ, Beckwith J. 1995. Tight regulation, modulation, and high-level expression by vectors containing the arabinose PBAD promoter. J Bacteriol 177:4121-30.

8. O'Loughlin CT, Miller LC, Siryaporn A, Drescher K, Semmelhack MF, Bassler BL. 2013. A quorum-sensing inhibitor blocks Pseudomonas aeruginosa virulence and biofilm formation. Proc Natl Acad Sci U S A 110:17981-6.
